# Confidence in subjective pain is predicted by reaction time during decision making

**DOI:** 10.1038/s41598-020-77864-8

**Published:** 2020-12-07

**Authors:** Troy C. Dildine, Elizabeth A. Necka, Lauren Y. Atlas

**Affiliations:** 1 National Center for Complementary and Integrative Health, National Institutes of Health, 10 Center Drive, Bethesda, MD 20892 USA; 2 Department of Clinical Neuroscience, Karolinska Institutet, 171 77 Solna, Sweden; 3 National Institute of Mental Health, National Institutes of Health, Bethesda, MD 20892 USA; 4 National Institute On Drug Abuse, National Institutes of Health, Bethesda, MD 20892 USA

**Keywords:** Psychology, Human behaviour, Health care

## Abstract

Self-report is the gold standard for measuring pain. However, decisions about pain can vary substantially within and between individuals. We measured whether self-reported pain is accompanied by metacognition and variations in confidence, similar to perceptual decision-making in other modalities. Eighty healthy volunteers underwent acute thermal pain and provided pain ratings followed by confidence judgments on continuous visual analogue scales. We investigated whether eye fixations and reaction time during pain rating might serve as implicit markers of confidence. Confidence varied across trials and increased confidence was associated with faster pain rating reaction times. The association between confidence and fixations varied across individuals as a function of the reliability of individuals’ association between temperature and pain. Taken together, this work indicates that individuals can provide metacognitive judgments of pain and extends research on confidence in perceptual decision-making to pain.

## Introduction

Pain is a subjective experience, yet patients must engage in decision making and translate their internal experience to a verbal descriptor in order to obtain treatment and relief. Decades of work have focused on pain psychophysics and factors that modulate pain^1–6^ but few studies have investigated the pain decision process itself^7–10^. Understanding factors that guide pain decision making is essential, as clinicians and researchers continue to rely on unidimensional methods to assess patients’ pain (e.g., a visual analogue scale or verbal pain report). These methods assume that pain is constructed consistently within and across individuals; however this assumption is unlikely, as research has shown uncertainty affects decision making in other sensory modalities (e.g.,^11^) and manipulating uncertainty affects subjective pain^12,13^. Although recent work has assessed confidence in decisions comparing multiple nociceptive stimuli^14^, to our knowledge no studies have investigated how individuals judge certainty or confidence in their subjective pain ratings. To address this gap, we measured whether individuals can provide metacognitive insights on their pain and whether implicit measures predict explicit subjective uncertainty (i.e., lack of confidence) in pain ratings.

Determining whether individuals are capable of pain metacognition is critical to understanding the pain decision-making process. Metacognition is described as ‘knowing about knowing’^15^ and involves judgments about one’s decisions or inferences about one’s knowledge^16–18^. While we have a growing understanding of the metacognitive processes that guide decision making in domains as varied as memory^19^, value-based decisions^20^, and multi-sensory decision making^21^, we know relatively little about how individuals make inferences about their pain. In other domains, metacognitive judgments are tied to internal factors such as introspection^22^ and to external factors such as the magnitude of sensory information that is received^23,24^. Similarly, pain is intrinsically linked to both internal states (e.g., attention, anxiety, and expectations) and external factors (e.g., objective intensity of the noxious stimulus and predictive cues). The question of whether metacognition accompanies pain similarly to other modalities is largely unknown. People may exhibit variance in judging how their pain relates to the intensity of an objective noxious stimulus, and how confident they are in the pain ratings themselves. Alternatively, if people cannot introspect in their subjective pain (e.g., if pain ratings are themselves the “gold standard”^25^) there may be no meaningful variation in meta-cognitive reports.

Recently, Beck and colleagues^14^ provided the first test of metacognition of noxious stimulation. Participants compared an objective reference stimulus (i.e., a specific temperature) with a target stimulus of varying intensity (i.e., a different temperature) and reported which stimulus was more painful; this provided a measure of pain-related accuracy. Participants provided similar judgments about visual stimuli and made metacognitive judgments of confidence in their decisions about both types of stimuli. Metacognition acted similarly across sensory domains: pain judgments were similar to judgments of visual stimuli in overall confidence, metacognitive efficiency (confidence given a certain level of performance or ability to process a signal), and metacognitive sensitivity (confidence-accuracy correlation). However, individual differences in metacognitive efficiency and metacognitive sensitivity were not correlated across domains (i.e. some participants had better metacognitive sensitivity for pain, while others had better sensitivity for vision), suggesting distinctions between subjects. This study provided a critical first step in indicating that individuals can make metacognitive inferences about acute noxious stimulation. However, there are important distinctions between categorizing noxious stimuli and subjective pain. Pain is fundamentally distinct from nociception, the actual encoding of potentially damaging stimuli^26^. While an individual can rate which of two stimuli *feels* more painful, a task that measures accuracy in comparisons based on *objective* stimulus intensity is actually measuring nociception rather than pain, which is a subjective experience. Testing accuracy is important, as this provides a way to compare confidence with actual task performance, (i.e., metacognitive sensitivity, bias, and efficiency^16^). However, assessing responses solely based on stimulus intensity fails to incorporate the myriad of factors that lead to meaningful variations in pain even within the same objective stimulus intensity, such as sensitization^27^, habituation^28^, and variations in attention^29,30^. Pain researchers have discussed at great length the importance of trusting individuals’ pain ratings, rather than searching for objective measures that can invalidate patients’ pain^31^. This leaves open the question of whether people experience variable confidence in judgments about their subjective pain rating.

It is possible that many forms of pain modulation, including both psychological interventions (e.g. placebo) and pharmacological treatments, interact with not only pain but also confidence in one’s pain. For example, one must evaluate the intensity of a headache and one’s confidence in one’s symptoms when determining whether or not to take an over-the-counter analgesic. After taking medication and subsequently experiencing relief, one might attribute the relief to the pill, or one might reevaluate the initial headache and consider whether it might have subsided on its own. At this point, one might reevaluate one’s confidence in their initial headache intensity. To understand how confidence in pain might affect pain-related decision-making, we must first establish whether individuals are capable of pain metacognition and how to detect when an individual is experiencing uncertainty in pain.

**Figure 1:**
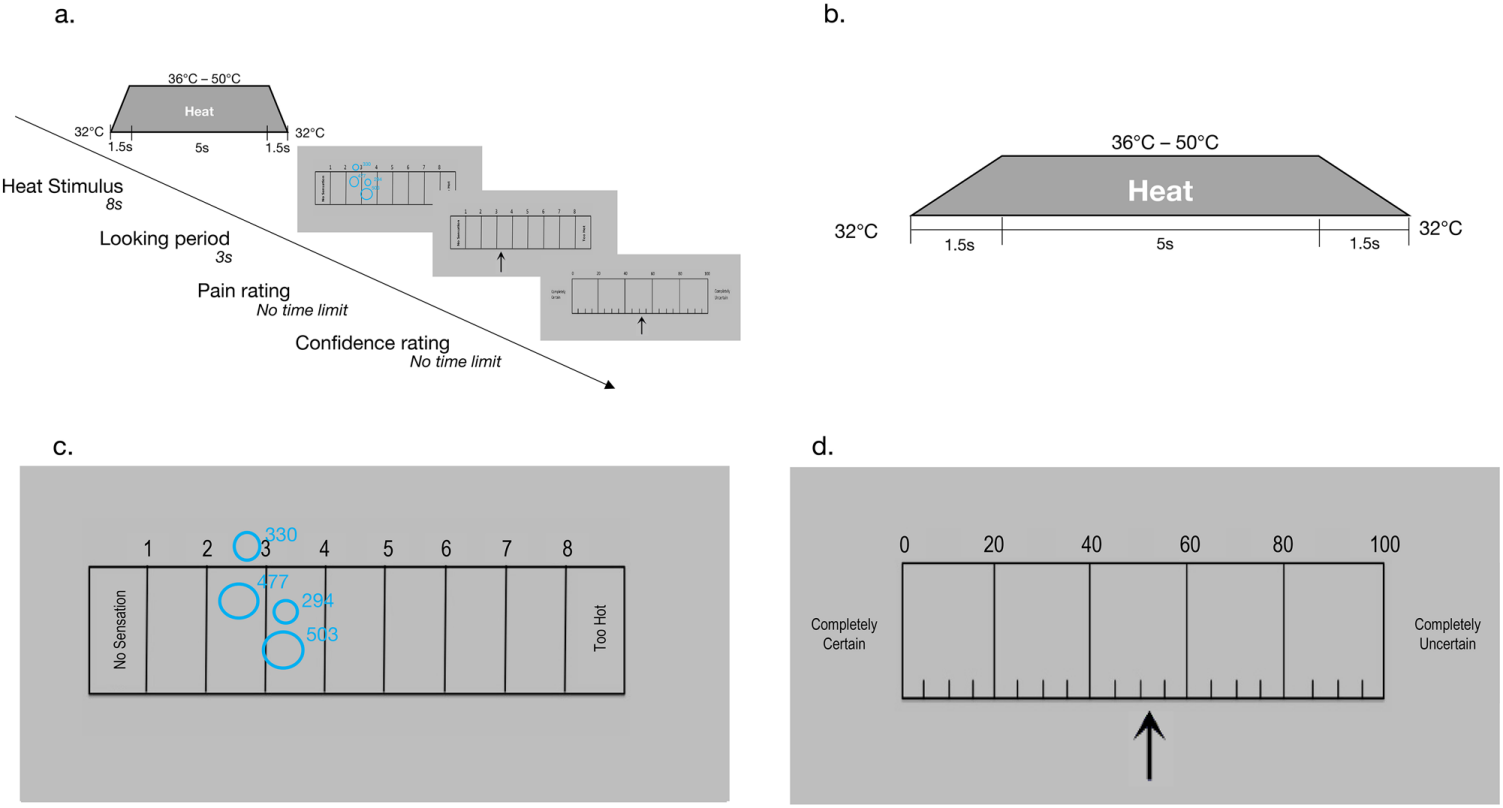
Task design. *A. Schematic of trial design.* Trials began with a Heat Stimulus, followed by a three-second looking period, during which participants could look at the pain rating scale (while their eye movements were tracked) but could not make a pain rating. After three-seconds of pain rating scale presentation, an arrow appeared on the scale and participants made a pain rating for the preceding stimulus. Finally, participants provided confidence ratings. There was no time limit for pain ratings or confidence ratings. *B*. *Heat stimulus.* Each 8-s heat stimulus included 5 s of stimulation at a peak destination temperature ranging from 36 °C to 50 °C, as well as 1.5-s ramps to and from a baseline of 32 °C. After 5 s at peak temperature, the stimulus ramped down to 32 degrees Celsius in 1.5 s. *C*. *Looking period.* Example of gaze position data during the Looking period, prior to pain rating. Each circle represents a fixation on the pain rating scale. Area and numbers denote the duration of the fixation, which was not used for the current analysis. *D*. *Confidence rating.* Following pain rating, participants rated their confidence using a visual analogue scale that ranged from “Completely Certain” to “Completely Uncertain”.

To probe whether individuals vary in confidence in their pain ratings, we applied acute noxious thermal stimulation to the volar forearm of healthy volunteers and measured self-reported pain and confidence in pain rating (see Fig. 1 for task design). Prior research indicates that less confident decisions are associated with slower reaction times^32,33^ and increases in the number of eye fixations on visual alternatives during decision-making ^34–37^, and that introspective accuracy varies substantially across individuals^22^. We therefore measured pain rating reaction time and the number of eye fixations participants made while viewing the pain scale, as well as the overall variance in a participant’s pain ratings that could be explained by temperature (i.e., the *reliability* of a participant’s temperature-pain relationship), which might relate to introspective accuracy.

Consistent with metacognition in other sensory modalities, we hypothesized that participants would exhibit variance in their confidence in their pain ratings and would take longer to make judgments about pain and exhibit more fixations when considering their pain (i.e., during pain rating scale presentation) when they were less confident. We hypothesized that these relationships would be stronger in individuals with greater reliability between pain rating and stimulus intensity. In light of evidence that confidence increases when there is more sensory information^38^ and as a function of experience^39^, we also hypothesized that confidence would increase as a function of noxious stimulation intensity and across time. If individuals make meaningful metacognitive judgments about pain and if such variance can be identified using explicit self-report and/or implicit behavioral measures, then future studies should measure metacognition to gain insight on how confidence might modulate pain and what factors shape pain-related confidence.

## Results

### Participants report variations in confidence about subjective pain

Eighty healthy volunteers experienced brief noxious thermal stimulation (*M*_temperature_ = 44.89 °C, *SD*_temperature_ = 3.15 °C) and rated their pain on a 0–10 visual analogue scale (VAS; *M*_pain_ = 4.58, *SD*_pain_ = 2.71) following a 3-s looking period, in which the scale was presented and we measured the number of fixations. Temperatures were iteratively updated using an adaptive calibration to elicit ratings of 2, 5, and 8 on the pain scale (see Methods). We fit an initial linear regression between temperature and pain rating from the first 3 heat stimulations. The fit was iteratively updated and used to predict the remaining 21 temperatures consistent with prior work using this approach (e.g.,^40–43^). Although all temperatures were estimated to evoke pain, some trials (*M*_*within-subjects*_ = 3.96 trials) were rated as non-painful. Analyses in the main manuscript include all trials, and we report results of analyses restricted to painful trials in Supplementary Results (see ‘Correlational analyses restricted to painful trials’; ‘Two-part model restricted to painful trials: Reaction time’; and ‘Two-part model restricted to painful trials: Number of fixations’). On average, much of the variance in participants’ pain ratings could be explained by the temperature of the stimulus they experienced (*M*_r2_ = 0.66, *SD*_r2_ = 0.17); we refer to the amount of variance in pain ratings explained by temperature (i.e., R^2^ of the pain-temperature association) as reliability.

**Figure 2:**
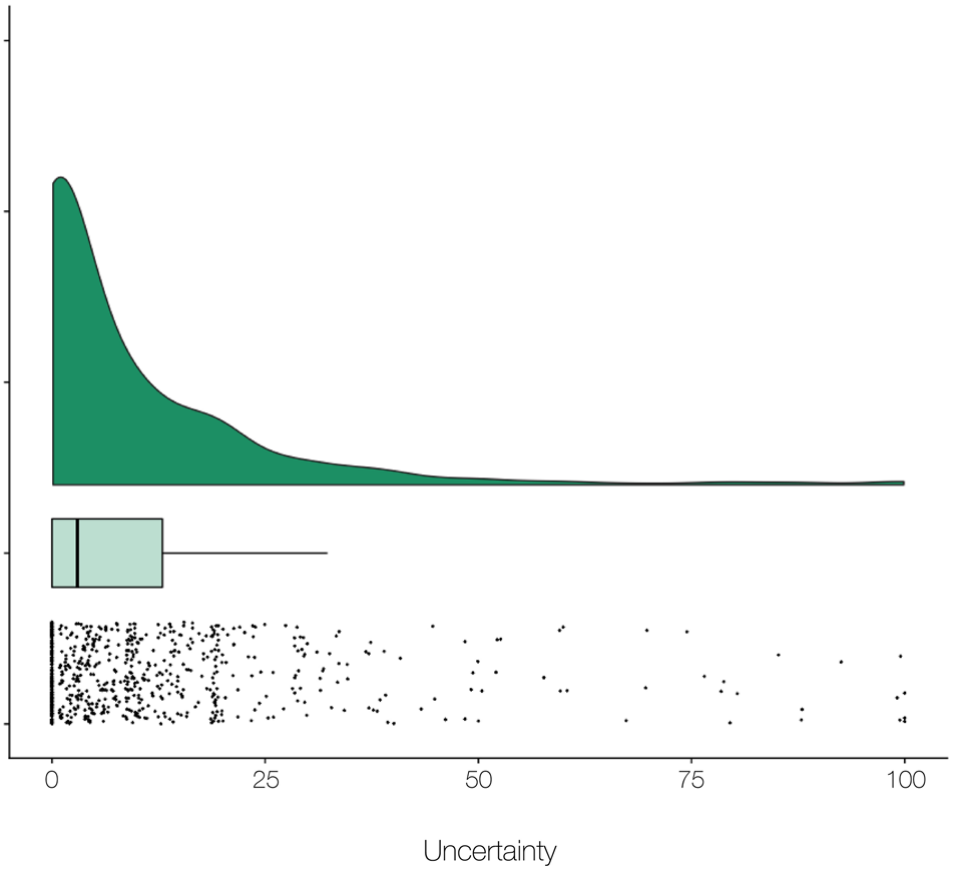
Distribution of uncertainty. This figure depicts uncertainty ratings across participants, smoothed via a kernel density function (top) and depicted as raw ratings (below). Uncertainty was present during the task (*M*_uncertainty_ = 9.48, *SD*_*uncertainty*_ = 15.63); however, the scores were zero-inflated confirmed via a zero-inflation score test based on a χ_1_^2^ distribution (*S*($\tilde{\beta}$) = 4,145,914, *p* < 0.001. We therefore used a two-part model to account for zero-inflated data (see Fig. 4).

Immediately after each pain rating, participants rated uncertainty in their pain rating for that trial using a 0–100 VAS, in which 0 denoted complete certainty and 100 complete uncertainty. Across all trials, participants reported low levels of uncertainty (*M*_uncertainty_ = 9.48, *SD*_*uncertainty*_ = 15.64, *Coefficient of Variation* = 165.0%), such that 817 trials (42.5%) were rated with zero uncertainty. Eight participtants (10%) only rated with zero uncertainty and were not included in any analyses assessing associations and effects of predictors on confidence. Still, participants on average reported non-zero uncertainty: a one-sample t-test on individual participant’s *W*, the test-statistic resulting from a within-subject Wilcoxon signed rank test on uncertainty responses for each individual, demonstrated that participants do experience some degree of uncertainty in their pain judgments (*M*_*W*_ = 2.14, *SD*_*W*_ = 2.05, *t*(75) = 9.09, *p* < 0.001, CI [1.67, 2.61]). In other words, participants are not entirely confident in their subjective pain ratings. However, the data overall were zero-inflated, meaning that there was a preponderance of trials on which participants reported complete confidence. This was confirmed via a zero-inflation score test based on a $\chi$_(1)_^2^ distribution^44^ (*S*($\tilde{\beta}$~)) = 4,145,914, *p* < 0.001; see Fig. 2 for a distribution of uncertainty). Uncertainty ratings did not differ by sex or race (all *p*’s > 0.1).

### Pain-related uncertainty decreases over time and is associated with slower reaction times during pain rating

**Figure 3:**
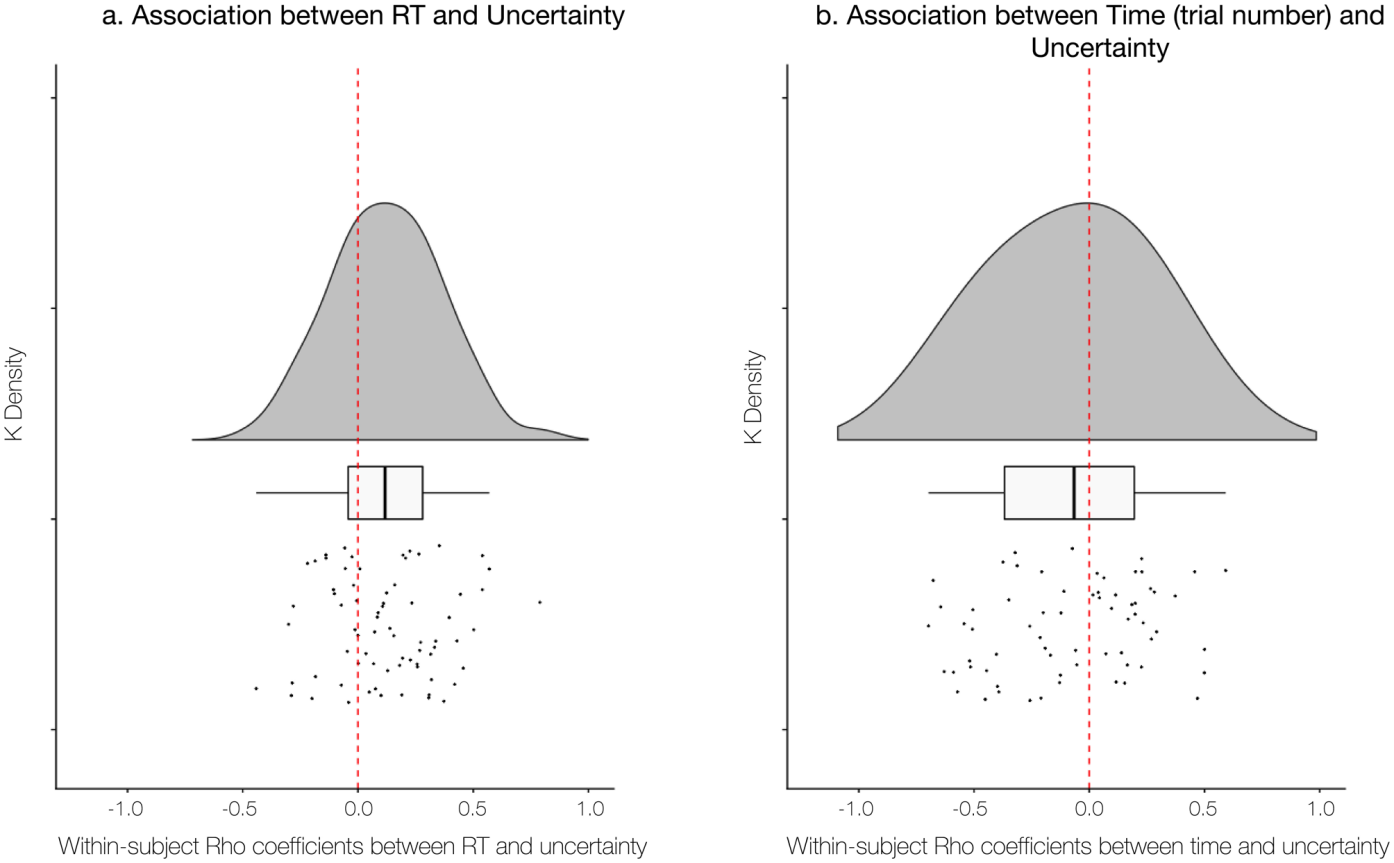
Distribution of within-subject associations between Reaction Time and Uncertainty & between Time and Uncertainty. Here we depict distributions of within-subject associations with uncertainty based on rho coefficients, smoothed with a kernel density function (top) with boxplot and individual subject’s rho coefficients below. Correlations were run between uncertainty and reaction time (RT) and between uncertainty and time across trials within each participant. We ran a one sample t-test against zero on the subject-level rho coefficients for each predictor respectively. (**a**) There was a positive association between uncertainty and reaction time (*M*_rho_ = 0.12, *SD*_rho_ = 0.24, *t*(71) = 4.38, *p* < 0.001, CI [.07, .18]) such that participants took a longer time to rate their pain when they reported more uncertainty. (**b**) There was a negative association between uncertainty and time (*M*_rho_ = − 0.08, *SD*_rho_ = 0.34, *t*(71) =  − 2.05, *p* = 0.04, CI [− 0.16, − 0.002], such that participants reported less uncertainty on later trials (i.e. with more experience in the task).

We used within-subjects Spearman’s correlations to investigate each individual’s associations between uncertainty (for those with variable uncertainty; n = 72) and each of our behavioral measures, as well as time (i.e., trial number) and sensory intensity (i.e., temperature) across 24 trials. The correlation between uncertainty and number of eye fixations was restricted to those with valid eye data (n = 66). We calculated rho coefficients for each individual and for each association and compared the distribution of rho values against zero using one-sample t-tests for each independent variable (find full results in Supplementary Table S1). We also computed the correlations restricted to painful trials and report our results in the Supplementary Results (see ‘Correlational analyses restricted to painful trials’). Furthermore, we investigated the association between reliability (one value per person) and mean uncertainty (one value per person) by running an across-subjects Spearman’s correlation. Across participants, there was a positive association between uncertainty and reaction time (*M*_rho_ = 0.12, *SD*_rho_ = 0.24, *t*(71) = 4.38, *p* < 0.001, CI [0.07, 0.18]; see Fig. 3a for a distribution of the rho coefficients^45^), such that participants took longer to rate pain when they were more uncertain. There was a negative association between uncertainty and time (*M*_rho_ = − 0.08, *SD*_rho_ = 0.34, *t*(71) = − 2.05, *p* = 0.04, CI [− 0.16, − 0.002]; see Fig. 3b for a distribution of the rho coefficients), such that individuals reported less uncertainty on later trials. We analyzed within-subject correlations between reaction time (RT) and time (trial number) and observed a significant difference from zero across rho coefficients (*M*_rho_ = − 0.30, *SD*_rho_ = 0.26, *t*(79) =  − 10.42, *p* < 0.001, CI [− 0.36, − 0.24]), such that reaction times decreased over the course of the experiment. Uncertainty was not consistently associated with number of fixations, reliability, or temperature (all *p*’s > 0.4). However, we note that uncertainty was negatively associated with temperature when we restricted analyses to painful trials (*M*_rho_ = − 0.09, *SD*_rho_ = 0.30, *t*(70) =  − 2.43, *p* = 0.02, CI [− 0.16, − 0.02]) such that uncertainty was greater at lower temperatures (full results in Supplementary Results: ‘Correlational analyses restricted to painful trials’). For each test, we also compared rho coefficient distributions by sex and race and observed no difference as a function of race or sex (all *p*’s > 0.1).

### Two part multilevel model: uncertainty is associated with slower reaction times during pain rating

**Figure 4:**
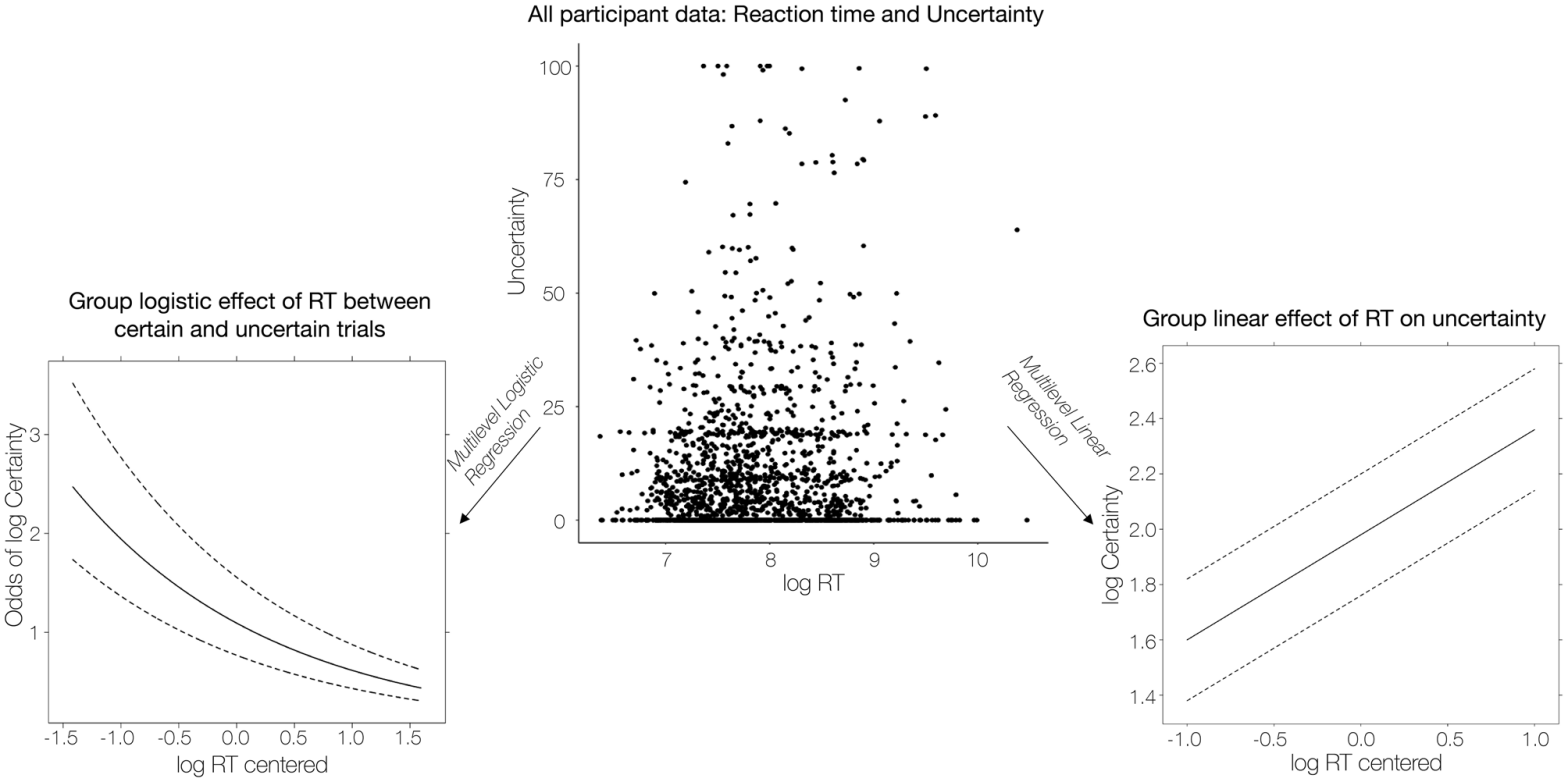
Associations between reaction time and uncertainty based on two-part multilevel model. *Top*: Confidence ratings were heavily weighted toward zero (i.e. complete certainty; see also Fig. 2). We therefore used a two-part multilevel model^43^ to measure associations with uncertainty. The first (logistic) part classified trials into either ‘trials with uncertainty’ OR ‘trials with no uncertainty’ across all participant while the second (linear) part tested associations with variations in uncertainty within non-zero (i.e. uncertain) trials. *Lower left:* In the logistic part of the model, we evaluated whether fixations or reaction time (reaction time is shown) predicted the likelihood that a trial was rated with uncertainty or not across all participants. The solid black line represents the group estimate (exponentiated to transform from log odds to odds) and the dashed lines represents the confidence interval. *Lower right:* The linear part of the model evaluated whether fixations or reaction time (reaction time is shown) predict the variations in uncertainty within uncertain trials. The solid black line represents the group estimate and the dashed lines represents the confidence interval.

The results above were based on one-sample t-tests of correlations within individuals, for comparison to previous work on metacognition using summary statistics approaches (e.g.,^33^). However, because trials were nested within subjects in our repeated-measures design, a more appropriate approach would be to use multilevel models that simultaneously model within- and between-subject factors. Because standard linear models were inappropriate for our zero-inflated uncertainty ratings (see Supplementary Methods: ‘Initial linear mixed models’), we used a two-part multilevel model^46–49^ on log-normal data (see Methods and Fig. 4). The first part of the model incorporated a logistic regression to predict trials rated with zero uncertainty versus trials on which participants reported some uncertainty (i.e., uncertainty > 0; irrespective of the magnitude of the non-zero rating). The second part consisted of a linear model that measured associations between behavioral measures and variations in log-transformed uncertainty on trials when participants reported any level of uncertainty in their pain rating. We ran a two-part model for reaction time and a separate two-part model for number of fixations; therefore, we applied a Bonferroni correction and set alpha to 0.025 for each model. For each model, we only incuded participants that had at least four trials rated with and without complete certainty (reaction time: n = 37; number of eye fixations: n = 35; see Methods ‘Analytic Strategy’ for a full breakdown).

We ran model comparisons to find the simplest and best-fit model by adding one fixed or one random effect at a time and determining if the model significantly improved. The simplest model for log-normal reaction time (reaction time in ms), which included a fixed and random intercept and reaction time as a fixed effect but did not include time, reliability or temperature provided the best model fit via likelihood ratio tests (find full model comparison in Supplementary Table S2). Our final model for log-normal reaction time was:

1 $$\left\{ {\begin{array}{*{20}l} {{\text{LINEAR}}} \hfill & {{\text{Uncertainty}}_{{{\text{ij}}}} > 0{ = (}\upgamma _{{{00}}} +\upgamma _{10} {\text{ReactionTime}}_{{{\text{ij}}}} ) + (u_{{0{\text{j}}}} ) + {\text{r}}_{{{\text{ij}}}} } \hfill \\ {{\text{LOGISTIC}}} \hfill & {{\text{In}}\frac{{{\text{Uncertainty}}_{{{\text{ij}}}} = 0}}{{{1} - {\text{Uncertainty}}_{{{\text{ij}}}} = 0}}{ = (}\upgamma _{{{00}}} +\upgamma _{10} {\text{ReactionTime}}_{{{\text{ij}}}} ) + (u_{{0{\text{j}}}} ) + {\text{r}}_{{{\text{ij}}}} } \hfill \\ \end{array} } \right.$$

We visualized the residuals from the two-part model via a qq-plot based on custom code (https://drizopoulos.github.io/GLMMadaptive/articles/Goodness_of_Fit.html) for the DHARMa package^50^ in R, and observed no deviations in the residuals (i.e., the model met assumptions; see visual in Supplementary Fig. S3).

**Figure 5:**
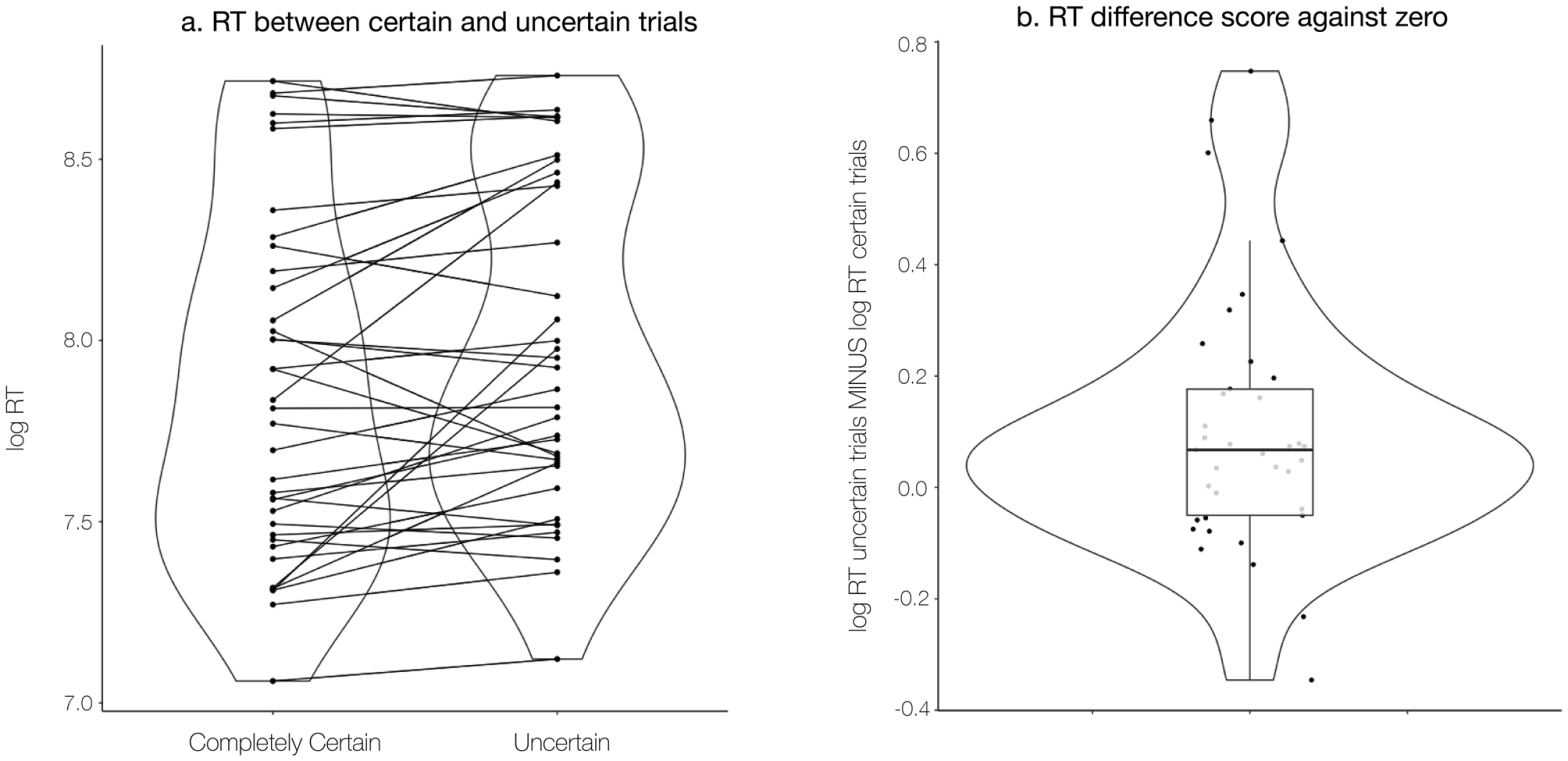
Differences in reaction time between certain and uncertain trials. Participants were quicker to rate pain during certain trials (*t*(36) = 2.70, *p* = .01, CI = [.03, 0.18], mean difference = 0.11 log RT (RT in ms)). (**A**) We used violin plots to present participant’s mean log reaction time during certain trials (left violin plot; *M*_*logRT*_ = 7.86 log RT, *SD*_*logRT*_ = 0.48) and uncertain trials (right violin plot; *M*_*logRT*_ = 7.96 log RT, *SD*_*logRT*_ = 0.45) and lines to indicate change in reaction between certain and uncertain trials for each participant. (**B**) We present participant difference scores (*M*_*difference score*_ = 0.10, *SEM*_*difference score*_ = 0.038), computed by subtracting the mean reaction time during certain trials from the mean reaction time during uncertain trials for each participant. A boxplot shows the median difference value (*Median*_*difference score*_ = 0.07) and interquartile range (*IQR* = 0.23; *Q1*: − 0.05, *Q3*: 0.18).

The logistic portion of the model revealed a negative effect of reaction time in predicting certainty (β_1Logistic_ = − 0.55, Odds: 0.58, *SEM* = 0.18, *z* = − 3.09, *p* = 0.002; see Fig. 5 and Table 1 for full model results) such that increasing log-normal reaction time by one log-unit (i.e., slowing the response) decreased the odds of being certain by 42%. In other words, longer reaction times were associated with lower odds of expressing complete certainty. The linear portion of the two-part model revealed a positive effect of log-normal reaction time on log-normal uncertainty (β_1Linear_ = 0.40, *SEM* = 0.11, *z* = 3.66, *p* < 0.001) such that slower ratings were associated with higher uncertainty.

**Table 1:** Asssociation between confidence and reaction time based on Two-Part Multilevel Models.

Variable	β	SE	z	*p*
Logistic				
Intercept	1.98	0.13	15.14	< 0.001
Reaction time	0.37	0.11	3.40	< 0.001
Linear				
Intercept	0.08	0.17	0.54	0.59
Reaction time	− 0.57	0.18	− 3.20	0.001

Statistical outcomes for the two-part multilevel models are reported separately for linear and logistic portions of the model.

As the two-part model is newly implemented in R^49^, we ran multiple models (see Supplementary Methods) with the same parameters to verify our results, including (1) a two-stage simple statistics approach^51^; (2) a multilevel linear model on non-zero uncertainty trials; and (3) a multilevel logistic model to compare certain and uncertain trials.

Findings from both the logistic and linear parts of the two-part model were consistent across different modeling approaches. In the two-stage simple statistics approach, one-sample t-tests revealed consistent associations across subjects for both individual logistic regression coefficients (*M*_logistic_ = 0.94, *t*(36) = 2.02, *p* = 0.051, CI [− 0.004, 1.88] and individual linear regression coefficients (*M*_Beta_ = 0.57, *t*(64) = 2.15, *p* = 0.04, CI [0.04, 1.11]), although we note that both were marginal after Bonferroni correction (for full results, see Supplementary Results: ‘Two-stage multilevel model: Reaction time’). Similarly, single-part multilevel models replicated findings from the two-part multilevel models. Our logistic multilevel model implemented in R’s glmer package revealed a significant association between reaction time and uncertainty on trials in which subjects were not completely confident (β_1Linear_ = 0.25, *SEM* = 0.05, *t* = 4.8, *p* < 0.001). For full results, see Supplementary Results: ‘Single-part multilevel linear and logistic models: Reaction Time’.

Finally, because we observed a significant association between time and reaction time in our correlational analyses, we ran an additional two-part model for reaction time which included mean-centered trial number (time) and an interaction term between reaction time and trial number. We observed no interaction between the two variables in either the logistic or linear portions and the effect of reaction time on both the linear and logistic portions of the model remained (for full details see: Supplementary Results: “Two-part model for reaction time: Including time and the interaction between time and reaction time as fixed effects*.”* ).

### Uncertainty decreases over time and association with number of fixations depends on reliability

We ran model comparisons and observed a model including a fixed and random intercept and fixed effects of number of fixations, time, reliability and an interaction term between reliability and number of fixations provided the best fit (see full model comparison in Supplementary Table S3). Our final model for number of fixations was:

2 $$\left\{ {\begin{array}{*{20}l} {{\text{LINEAR}}} \hfill & {{\text{Uncertainty}}_{{{\text{ij}}}} > 0{ = (}\upgamma _{{{00}}} +\upgamma _{10} {\text{FixationNumber}}_{{{\text{ij}}}} +\upgamma _{10} {\text{time}}_{{{\text{ij}}}} +\upgamma _{01} {\text{R}}_{{\text{j}}}^{2} +\upgamma _{11} {\text{FixationNumber}}_{{{\text{ij}}}}\upgamma _{01} {\text{R}}_{{\text{j}}}^{2} ) + (u_{{0{\text{j}}}} ) + {\text{r}}_{{{\text{ij}}}} } \hfill \\ {{\text{LOGISTIC}}} \hfill & {{\text{In}}\frac{{{\text{Uncertainty}}_{{{\text{ij}}}} = 0}}{{{1} - {\text{Uncertainty}}_{{{\text{ij}}}} = 0}}{ = (}\upgamma _{{{00}}} +\upgamma _{10} {\text{FixationNumber}}_{{{\text{ij}}}} +\upgamma _{10} {\text{time}}_{{{\text{ij}}}} +\upgamma _{01} {\text{R}}_{{\text{j}}}^{2} +\upgamma _{11} {\text{FixationNumber}}_{{{\text{ij}}}}\upgamma _{01} {\text{R}}_{{\text{j}}}^{2} ) + (u_{{0{\text{j}}}} ) + {\text{r}}_{{{\text{ij}}}} } \hfill \\ \end{array} } \right.$$

**Figure 6:**
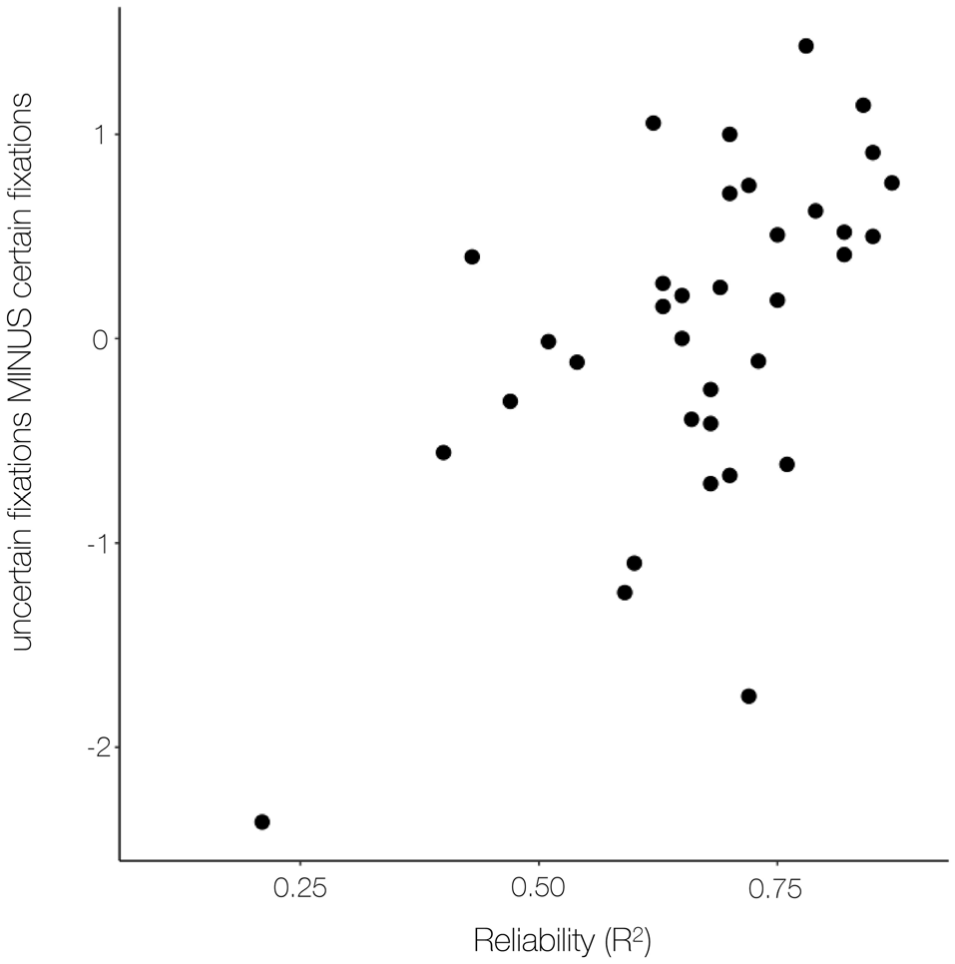
Association between reliability and the difference in fixations during certain and uncertain trials. For each participant, we computed a difference score by subtracting the average number of fixations during certain trials from the average number of fixations made during uncertain trials. The effect of certainty on fixations (y-axis) was significantly correlated with reliability (x-axis), the strength of the association between temperature and pain (*r* = 0.57, *p* < .001). This association is still present, although weaker, when the outlier near the graph’s origin is removed (*r* = 0.4, *p* = .02).

In the logistic portion of our model, we observed an interaction between number of fixations and reliability, β_1Logistic_ = − 1.12, Odds: 0.33, *SE* = 0.34, *z* = − 3.35, *p* < 0.001 (see a scatter plot between reliability and number of fixations difference score in Fig. 6), such that a one unit increase in number of fixations is associated with a 1% decrease in odds of being certain when reliability is average, but for every unit increase in reliability the effect of number of fixations on odds of being certain increases by 67% (see Table 2 for full results). In other words, for participants who exhibited greater reliability in their association between pain and temperature, more fixations were associated with higher odds of uncertainty, whereas for participants who exhibited less reliability, more fixations were associated with higher odds of certainty. There was no additional association between uncertainty and fixation in the linear part of the model (*p* > 0.1). There was no relationship with time in the logistic part of the model (*p* > 0.4). However, in the linear portion of our model, we observed a negative association between time and uncertainty (β_1Linear_ = − 0.02, *SEM* = 0.007, *z* = − 2.5, *p* = 0.01) such that participants reported more certainty later in the task.

**Table 2:** Association between confidence and number of fixations via Two-Part Multilevel Model.

Variable	β	SE	z	*p*
Logistic				
Intercept	0.75	0.17	0.44	0.66
Number of Fixations	− 0.01	0.05	− 0.22	0.83
R^2^	0.87	1.21	0.72	0.47
Time	0.008	0.01	0.74	0.45
Number of Fixations*R^2^	− 1.12	0.34	− 3.35	< 0.001
Linear				
Intercept	1.97	0.13	15.5	< 0.001
Number of Fixations	0.01	0.02	0.38	0.70
R^2^	0.28	0.89	0.32	0.75
Time	− 0.02	0.007	− 2.58	0.009
Number of Fixations*R^2^	0.26	0.18	1.43	0.15

Statistical outcomes for two-part multilevel models are reported separately for linear and logistic portions of the model.

The association between reliability and the uncertainty-fixation relationship from the logistic part of the two-part model was robust to different modeling approaches. Two-stage simple statistics revealed a significant association between reliability and individual logistic regression coefficients and reliability (β_1Linear_ = 1.88, *SEM* = 0.50, *t*(32) = 3.8, *p* < 0.001; for full results, see Supplementary Results: ‘Two-stage multilevel model: Number of fixations’). We also observed a significant interaction with reliability in the single-part logistic multilevel model implemented with glmer (β_1Logistic_ = 1.09, Odds: 2.97, *SEM* = 0.33, *z* = 3.3, *p* = 0.001). For full results, please see Supplementary Results: ‘Single-part multilevel linear and logistic models: Number of fixations’.

The negative association between time and uncertainty from the linear part of the two-part model was also robust across modeling approaches: it was replicated in both the two-stage simple statistics approach via a one-sample t-test on individual linear regression betas (*M*_Beta_ = − 0.03, *t*(60) = − 2.9, *p* = 0.005, CI [− 0.05, − 0.009]) and in the linear multilevel model via lmer (β_1Linear_ = − 0.02, *SEM* = 0.004, *t* = − 4.2, *p* < 0.001).

## Discussion

Our results extend the literature of metacognition and perceptual decision making to decisions related to pain. Individuals experienced variations in confidence when rating acute pain, suggesting that individuals can make metacognitive judgments about their pain experience and are aware of their confidence (or lack thereof). We observed mixed results for associations between confidence and our behavioral measures, number of fixations and reaction time. Similar to other modalities, reaction time was linked to confidence in pain, such that individuals were slower to rate pain on trials when they felt less certain. The association between confidence and number of fixations on the scale was less consistent across individuals, and varied as a function of the reliability between temperature and pain, providing a link between pain metacognition and introspective accuracy. Here we discuss the implications of these findings and future directions for this work.

Our findings indicate that pain metacognition is consistent with metacognition in other modalities in several ways. Pain rating reaction times decreased as confidence increased (i.e., individuals were faster to rate their pain when they were more confident) and confidence in pain ratings increased over time (i.e., individuals were more confident about their pain ratings as they gained experience with the noxious stimulation and made more metacognitive judgments about their pain). The effect of experience is particularly relevant for pain studies, which should implement practice trials or calibration procedures to increase the likelihood an individual will have confident pain reports during the study paradigm. Furthermore, it suggests the importance of including experience (e.g., trial number or time in task) as a covariate in repeated-measure study designs. Pain rating reaction time was associated with both whether or not an individual was confident on a given trial and their level of confidence. This effect exists even though differences in reaction time may have been diminished in our task, as we probed pain ratings after heat offset, and decisions about pain may occur concurrently with stimulation or while viewing the pain rating scale prior to recording a response. Furthermore, these results were robust to several different modeling options.

Pain metacognition also differed from other modalities in several ways. First, confidence was not associated with stimulus intensity across all trials (i.e., individuals did not have increased confidence on trials with higher temperatures). However, when we restricted to painful trials, higher temperatures, which are associated with greater nociceptor activation^52^, were positively associated with confidence. Second, confidence was only associated with the number of fixations made to the pain rating scale when accounting for reliability, in contrast to previous work demonstrating associations between fixations and confidence across participants in studies of visual perception and memory-related decision making (e.g.,^34,37^). The lack of associations between confidence and number of fixations across participants suggests the number of fixations made while viewing a pain rating scale is not a reliable marker of confidence in pain ratings across participants. We only measured fixations during a three-second looking period while participants viewed the pain scale prior to recording responses. It is possible that fixations would be more variable during the heat stimulation period itself; however, we did not display the pain rating scale during this period to allow for pupil dilation analyses in a larger study assessing physiological responses of pain^43^. Furthermore, it is possible that participants may have disengaged from the task and behaviors were diminished during the three-second looking period, as this period preceded mouse presentation used for pain ratings. However, we did find that the association between confidence and fixations was moderated by reliability. Although this was only present in the logistic portion of the model, it was robust to different modeling options, and suggests individuals with greater reliability exhibit more fixations when they are less confident, consistent with other domains of decision-making, whereas individuals with lower reliability exhibit *more* fixations when confident. This suggests the utility of eye fixations as an implicit marker of confidence in pain may be restricted to those with heightened sensory acuity, which is closely related to introspective accuracy. Studies that use calibration tasks to exclude participants with low sensitivity or reliability (which was a secondary goal of the pain task we used here) would likely find a stronger association between fixations and confidence across participants that mimics other modalities.

Participants reported high confidence in their pain overall, although confidence varied from trial to trial. In other sensory modalities confidence and perceptual awareness tends to be high when an individual is ‘correct’ on a trial^14^. Although pain has no objective ‘truth’, it is feasible that individuals deemed their subjective experience as correct responses (i.e., the pain the person felt is what they reported), hence why subjective confidence reports seem to mimic distributions of confidence found for ‘correct’ trials in other studies.

It is important to note that our study paradigm differed from most perceptual decision making tasks in several important ways. Many studies of confidence restrict the number of responses in their confidence scale (e.g., four discrete choices compared to a 0–100 continuous scale), which may lead to more variable decisions and prevent the zero-inflation we observed in our data. Furthermore, we note that participants may rate confidence towards the scale’s anchors (positioned at the extremes of our scale) and may generally overestimate their confidence^16^. The propensity to overestimate confidence is often assessed via metacognitive bias in the literature^16^. However, as our paradigm lacks an absolute truth (i.e., pain is inherently subjective, and therefore there is no objective, external marker to code accurate response) and we utilized a continuous, visual analogue scale for our pain rating decisions (opposed to a two-alternative forced choice design), it was not possible to assess whether our participants exhibited a metacognitive bias. Future research should also evaluate whether confidence and pain metacognition are impacted differently when subjects are asked to focus on sensory or affective aspects of pain (i.e. pain intensity versus pain unpleasantness) or when stimuli are described in terms of noxious stimulus intensity versus subjective pain (e.g. anchoring judgments on “too hot” versus “worst pain imaginable”).

We used an adaptive task that was restricted to painful stimulation and iteratively fit each participant’s pain sensitivity profile to identify their pain threshold and tolerance and determine each participants’ reliability. Tasks that use a wider range of stimuli, including painful trials, non-painful trials, and trials near the perceptual threshold, are likely to elicit larger variations in confidence. Furthermore, our participants provided pain ratings immediately after stimulus offset, and then provided confidence judgments. Although participants did not make decisions until after the heat stimulus subsided, it is possible that decision-making processes are most relevant during stimulation as participants gather evidence about the heat. Indeed, previous work has indicated that drift diffusion models can predict pain reports^53,54^, and that pain is associated with variations in both starting point and evidence accumulation. Future studies should evaluate whether on-line behavioral measures collected concurrent with noxious stimulation might be better predictors of confidence than those associated with post-stimulus ratings. Finally, we did not explicitly manipulate uncertainty during this task, which may have led to inflated confidence or decreased the likelihood that participants even engaged in metacognitive processes. Future studies should measure how experimental manipulations of uncertainty (e.g.,^12,13^) influence pain-related confidence and whether fixations and reaction time predict metacognitive judgments in cases where confidence is expected to vary more strongly within participants over time.

In summary, our findings suggest confidence can be measured during pain decision making and that individuals do experience varying levels of confidence in pain report. This suggests that individuals are not always confident about the pain they report, and they are cognizant of this fact. Future research on pain and pain modulation should incorporate confidence ratings to further understand features that drive confidence and uncertainty in pain, whether behavioral indicators predict subjective judgments, and to measure whether pain metacognition interacts with other forms of pain modulation. Analgesics, placebos, and other forms of pain modulation may have dissociable effects on pain and pain metacognition; for example, placebos might decrease pain while increasing uncertainty. Likewise, patients and participants who are high in metacognitive sensitivity may respond differently to interventions from participants with low metacognitive sensitivity. Understanding these additional features of pain decision making will improve our ability to tailor pain treatment and address all aspects of patients’ pain, including confidence in their pain decision-making.

## Methods

### Participants

Eighty healthy adult volunteers (*M*_*age*_ = 28.4 years old; *SD*_*age*_ = 7.9 years; 56% female; 35% White, 42% Black, 10% Asian, 8% Hispanic/Latino, 2% Two or more races, 3% unknown) provided informed consent in accordance with the Declaration of Helsinki and as approved by the National Institutes of Health (NIH) Combined Neuroscience IRB (Protocol 15-AT-0132). All participants were recruited via flyers placed on the NIH campus, through emails distributed to approved list servers, website postings (ClinicalTrials.gov: *NCT02446262*), or through the NIH patient recruitment office. Participants were not eligible if they had a history of chronic pain, neurological or psychiatric disorders, conditions affecting pain sensation or somatosensation, dermatological conditions on the volar forearm, or medication use that could affect pain sensation. Furthermore, participants were excluded if they had recent recreational drug use or were pregnant (both verified by urine sample). All participants were fluent in English and were determined to be in good health based on a nursing assessment prior to the task. Eligible participants underwent sensory testing to determine their eligibility for future studies. Participants received monetary compensation for their time and for receiving painful stimulation. Pain reports and heat-evoked physiological responses from this sample were included as a subset of a larger sample in a previous publication^43^ that did not evaluate confidence. Summary statistics were conducted on the full sample. Analyses assessing associations and effects of predictors on confidence were limited to 72 individuals (8 participants rated every trial with 100% complete confidence; i.e., they had no variability in the outcome measure) and to 66 individuals when assessing eye fixations (7 participants who failed our eye calibration, including 1 individual who failed the eye calibration but also reported no variability in their confidence).

### Stimuli and apparatus

Healthy volunteers experienced 24 trials of noxious thermal stimulation, which was applied to 8 skin sites on the non-dominant volar forearm, with 3 stimulations per site. Noxious thermal stimulation was applied using a 16 mm × 16 mm ATS thermode (Medoc Ltd., Ramat Yisha, Israel) attached by Velcro. We used the Eyelink 1000 Plus eye tracking system (SR Research Ltd., Ontario, Canada) to measure eye position and pupil dilation in all participants. Visual stimuli were presented using Experiment Builder software (SR Research Ltd., Ontario, Canada), which synchronized with the eye-tracking system to allow for precise task timing and measurements. Participants provided pain and uncertainty ratings using a computer mouse. The resolution of the screen was 1920 × 1080 with a refresh rate of 144 Hz. We used a chin rest to prevent the head from moving excessively. Participants were seated in a chair without wheels that was adjusted vertically to position the participant’s head at the chin rest. The chair was set 84 cm from the screen and distances from the participants eye to the top and bottom of screen were 52 and 58 cm respectively. We collected additional autonomic measures (skin conductance, heart rate, respiration, electrocardiogram) via the Biopac MP150 system (Biopac Systems Inc., Goleta, CA, USA), which were previously reported as part of a larger sample^43^ and are outside the scope of this paper. Analyses were conducted using Matlab 2019a (Mathworks Inc., Natick, MA), R version 3.6.3^55^ and R Studio 1.2.5033 (Boston, MA).

### General procedures

Participants provided consent and were escorted to the behavioral testing room. Prior to noxious stimulation, participants were familiarized with the thermode, rating scales, and completed state and trait questionnaires not analyzed here. They were then situated in the chair and the head rest was adjusted to an appropriate height to stabilize the head and maximize eye data quality. Once the participant was comfortable, the lens was focused, and the right eye was calibrated using a 9-point calibration. We validated the calibration and proceeded to the task when we achieved less than 1 degree of difference between calibration and validation for each site.

Participants underwent 24 heat stimulations of varying intensity on eight skin sites using an adaptive calibration procedure^40,43^ described below. Most studies assessing confidence in decisions utilize two-alternative forced choice designs and use adaptive staircases to adjust performance and maintain a criterion performance level (e.g.,^56^). Here, our adaptive calibration differed from other work on perceptual judgments in that we used an iterative regression procedure to target temperatures required to elicit ratings of low (2), medium (5), or high pain (8), rather than assessing a specific performance criterion. We discuss this approach in more detail below. To increase real-world application^57^ we used a continuous 0–10 pain scale to rate pain from a single stimulus and a continuous scale to probe confidence in pain judgments. Participants were instructed to provide ratings on a VAS ranging from 0 (no sensation) to 10 (most pain imaginable) using the following anchors: 1 = warm sensations; 2 = beginning of pain; 5 = moderate pain; 8 = most tolerable pain; 10 = worst pain imaginable (for exact language see Supplemental: ‘Pain rating scale instructions’). Participants were also told they could use decimals and if a participant found a stimulus to be intolerable, they were told that they could ask the experimenter to stop the stimulus immediately or they could remove the thermode from their arm. The participant was asked to rate this pain above an 8 on the scale (written as ‘Too hot’ on the scale) and this temperature was not applied to the same site on any subsequent trial, in order to avoid applying any temperatures that exceeded a participant’s tolerance (per IASP’s, *Ethical guidelines for pain research in humans*^58^). 98 trials were rated above 8 in the current study (M_within-subjects_ = 1.2 trials).

The first 3 temperatures were the same for all individuals (41 °C, 44 °C, and 47 °C). An initial linear regression between temperature and pain rating was created from the first 3 heat stimulations and was iteratively updated and used to predict the remaining 21 temperatures. The temperature applied on each trial was estimated to elicit one of three target pain intensities on the scale: pain threshold (rating of 2), medium pain (5), or pain tolerance (8). Each skin site was stimulated once at each target pain level. If a pain rating deviated from the estimate, then the line of best fit and the estimated temperatures for each target would update according to the degree of deviation (i.e., the slope and intercept would update).We used r^2^ to measure the reliability between stimulus temperature and subjective pain rating and included reliability as a moderator in our across-participants analyses. This measure also served as screening criteria for eligibility in future studies in our lab (participants with r^2^ < 0.4 did not continue to subsequent experiments).

During the task, each trial began with a black fixation box that appeared in the center of the screen. Participants were required to fixate on the cue for 500 ms in order for the task to advance. We marked the failure to fixate and manually advanced and repeated eye calibrations on trials when the participant was not able to fixate on the visual cue for 500 ms. The remaining trial elements are illustrated in Fig. 1. After cue offset, an eight-second heat stimulus was applied (1.5 s ramp to target temperature, 5 s at peak, 1.5 s ramp to baseline; the first 12 participants experienced ten-second heat stimuli with 7 s at peak). Following heat offset, a 1398-pixel wide (72.8% screen width) pain rating scale appeared for three seconds and participants were instructed to think about their rating while eye fixations were recorded. After three seconds, an arrow appeared at the center of the scale. Participants used the arrow to mark and rate their pain in addition to verbally confirming pain report. There was no time limit for this decision, and we measured pain rating reaction time (from the appearance of the arrow) on every trial.

Following the pain rating, participants rated how uncertain they were in their pain rating using a 0–100 scale, where 0 = completely certain and 100 = completely uncertain. We provided two questions to ensure proper usage of the uncertainty scale (see Supplementary Methods: ‘Instructions for rating explicit uncertainty’). We recognize that our scale probed confidence and not certainty^17,18^; therefore, we use the term confidence throughout the introduction and discussion of our manuscript, but we have kept the terms ‘certainty/uncertainty’ in our results as this more aptly relates to the instructions and scale used by participants. The uncertainty scale was aligned 200 pixels higher on the screen compared to the pain rating scale and anchored from completely certain to completely uncertain to prevent orienting or automatic carry-over from pain ratings. There was no time limit for the uncertainty ratings, and reaction time for uncertainty ratings was not analyzed further.

After each uncertainty rating was recorded, a prompt appeared that instructed the experimenter to move the thermode to the next skin site. The experimenter moved the thermode to the next skin site and began the next trial when the participant was ready.

### Data processing

Data was processed using Eyelink 1000 PLUS software (SR Research, 2009), which defines saccades as any period during which the eye exceeded a velocity of 30°/second or an acceleration of 8000°/second^2, and codes any period during which pupil and corneal reflection were tracked that was immediately preceded by or followed by a saccade or blink as a fixation (i.e., a moment when the retina is relatively stable on an item in the environment^59^). Eye data was exported from Eyelink into Matlab with custom code (https://github.com/djangraw/GazeVisToolbox). Based on recommendations from Holmqvist^60^, we excluded all fixations under 50 ms as well as fixations under 120 ms that are either preceded by or come right after a blink. Finally, triggers that marked the beginning and end of the 3-s looking period were used as event markers. We measured the number of valid fixations during this period on each trial for use in analyses irrespective of position. We chose to focus on the total number of fixations during the looking period, rather than restricting fixations to the scale or measuring gaze position, because our pain scale numerical anchors were above the scale and the mouse appeared on a restricted horizontal line below the scale, and because participants always fixated at the center of the screen during heat stimulation prior to pain scale presentation. Participants who had fewer than three trials of eye data or failed our fixation manipulation check were not included in analyses that included eye fixations (n = 7). For those individuals included in the final analyses with eye data (n = 66), trials with less than 50% eye data present during the three second looking period were excluded from analyses (*M* = 0.47 trials excluded per participant).

Pain and uncertainty ratings were transformed from raw pixel values to appropriate ratings by accounting for the screen position of the visual analogue scale. If an uncertainty rating was coded below zero or above 100 (i.e. the participant clicked slightly to the left or right of the scale), the mouse position was verified, and the rating recoded as zero or 100 accordingly (see Supplemental Methods: ‘Corrections for confidence ratings below 0 and above 100′). A rating of zero was of particular interest for our analyses, as we used a logistic regression to determine zero vs. non-zero confidence data (see Analytic Strategy below for more details).

We measured reaction time for the pain rating on each trial. To remove extreme outliers prior to our analyses, we applied minimal a-priori trimming per Baayen and Milin^61^ to our reaction time data. We first removed trials in which reaction time was less than or more than three standard deviations from the mean within-subject (*M* = 0.62 trials per participant) and then log-transformed to normalize the data.

### Analytic strategy

We began by running the Lilliefors composite goodness-of-fit test on uncertainty ratings via the lillietest function in Matlab to determine if our uncertainty data was normally distributed. Our data were not normal; therefore, we ran non-parametric versions of tests where normality is assumed. We ran non-parametric Wilcoxon signed rank tests to determine if uncertainty was reported differently from zero within subjects and ran a one-sample t-test against zero on the individual test scores, *W*. We ran Spearman’s correlations to evaluate associations between uncertainty and our independent variables (time, temperature, number of fixations, and reaction time). We examined rho coefficients across all subjects and tested whether rho coefficients were significantly different from zero via one-sample t-tests. We also investigated the association between reliability and confidence, but as we had only one value per participant for reliability (r^2^) we ran a single correlation (with mean confidence values) and report one rho coefficient across participants for this independent variable.

As independent and dependent variables were measured over time, within individuals, we next evaluated mixed models. We separately modeled confidence as a function of response time or number of fixations. We included reliability and its interaction with our variables of interest (reaction time and number of fixations) as we assumed potential impacts of perceptual awareness on metacognition and we included the external factors of time (trial number) and temperature as fixed covariates of interest. We initially evaluated linear mixed models on raw data followed by models using log and square root transformed uncertainty data (see Supplementary Methods: ‘Initial linear mixed models’), but determined that the residuals were not normally distributed and that confidence ratings were zero-inflated. To account for the high propensity of ‘completely certain’ responses, we ran a two-part mixed effects model^48^ to separate zero (i.e. 100% certainty) and non-zero data. The two-part model provides a critical method for analyzing metacognition for pain and subjective experiences. The model allowed us to jointly test 1) whether behaviors differ categorically between certain and uncertain trials and 2) whether behaviors are linearly associated with changes in uncertainty. We ran our two-part model using the GLMMadaptive package (version 0.6.8) in R^49^. To ensure sufficient variability to calculate log-odds for the logistic regression, we only included participants who had at least four trials with complete certainty and at least 4 trials with some uncertainty (Reaction time: n = 37 with 28 participants excluded due to too few certain trials and 7 participants excluded due to too few uncertain trials; Number of fixations: n = 35 with 26 participants excluded due to too few certain trials and 5 participants excluded due to too few uncertain trials).

Each two-part model treated uncertainty as a dependent variable and included an overall intercept as well as random intercepts for each participant. We created separate models for fixation number and reaction time. We added fixed and random effects, one at a time, to our model and computed likelihood ratio tests to determine whether a parameter should be included in the model. We ran likelihood ratio tests for each predictor to identify the simplest model with best fit and Bonferroni-corrected alpha levels to 0.025 as we used two separate models to find an implicit marker for confidence (see Supplementary Tables S2 and S3 for model comparisons for reaction time and number of fixations respectively). The simplest model for log-normal reaction time provided the best model fit via likelihood ratio tests (see ‘Model 1′ in Supplementary Table S2). Our final model for log-normal reaction time was:

1 $$\left\{ {\begin{array}{*{20}l} {{\text{LINEAR}}} \hfill & {{\text{Uncertainty}}_{{{\text{ij}}}} > 0{ = (}\upgamma _{{{00}}} +\upgamma _{10} {\text{ReactionTime}}_{{{\text{ij}}}} ) + (u_{{0{\text{j}}}} ) + {\text{r}}_{{{\text{ij}}}} } \hfill \\ {{\text{LOGISTIC}}} \hfill & {{\text{In}}\frac{{{\text{Uncertainty}}_{{{\text{ij}}}} = 0}}{{{1} - {\text{Uncertainty}}_{{{\text{ij}}}} = 0}}{ = (}\upgamma _{{{00}}} +\upgamma _{10} {\text{ReactionTime}}_{{{\text{ij}}}} ) + (u_{{0{\text{j}}}} ) + {\text{r}}_{{{\text{ij}}}} } \hfill \\ \end{array} } \right.$$

A model including reliability and time provided the best fits for a model predicting log-normal uncertainty via number of fixations (see ‘Model 4′ in Supplementary Table S3). Our final model for number of fixations was:

2 $$\left\{ {\begin{array}{*{20}l} {{\text{LINEAR}}} \hfill & {{\text{Uncertainty}}_{{{\text{ij}}}} > 0{ = (}\upgamma _{{{00}}} +\upgamma _{10} {\text{FixationNumber}}_{{{\text{ij}}}} +\upgamma _{10} {\text{time}}_{{{\text{ij}}}} +\upgamma _{01} {\text{R}}_{{\text{j}}}^{2} +\upgamma _{11} {\text{FixationNumber}}_{{{\text{ij}}}}\upgamma _{01} {\text{R}}_{{\text{j}}}^{2} ) + (u_{{0{\text{j}}}} ) + {\text{r}}_{{{\text{ij}}}} } \hfill \\ {{\text{LOGISTIC}}} \hfill & {{\text{In}}\frac{{{\text{Uncertainty}}_{{{\text{ij}}}} = 0}}{{{1} - {\text{Uncertainty}}_{{{\text{ij}}}} = 0}}{ = (}\upgamma _{{{00}}} +\upgamma _{10} {\text{FixationNumber}}_{{{\text{ij}}}} +\upgamma _{10} {\text{time}}_{{{\text{ij}}}} +\upgamma _{01} {\text{R}}_{{\text{j}}}^{2} +\upgamma _{11} {\text{FixationNumber}}_{{{\text{ij}}}}\upgamma _{01} {\text{R}}_{{\text{j}}}^{2} ) + (u_{{0{\text{j}}}} ) + {\text{r}}_{{{\text{ij}}}} } \hfill \\ \end{array} } \right.$$

As packages for two-part multilevel models of semi-continuous data are relatively new in R, we verified our results by running a simple summaries (two-stage) approach to the multilevel model (see Supplementary Methods: ‘Two-stage simple statistics approach to the two-part multilevel model’) and by running separate linear and logistic multilevel models with commonly used functions part of the lme4 package (v1.1–23;^62^) on the associated data (see Supplementary Methods: ‘*Validating two-part model with separate single-part multilevel linear and logistic models*).

Note that the logistic regressions used in models 1 and 3 for verification predict the odds of being non-zero (uncertain) whereas the logistic part in the two-part model predicts the odds of being zero; thus, the signs of the beta coefficients conflict across the models even when the implications of the beta coefficients within the context of its model are consistent. Comparisons to the two-part model are reported in Results and full results for each of the models can be found in Supplementary Results.

## Supplementary information

Supplementary Information.

## Data Availability

Behavioral data from participants who consented to data sharing (n = 46) is available on OSF^33^ at: https://osf.io/s46pr/. Eye data and analysis scripts are available upon request. *Custom code* The GLMMadaptive package used to run the two-part mixed effect model for semicontinuous data can be found at: https://cran.r-project.org/package=GLMMadaptive and the custom code to verify model assumptions can be found at: https://drizopoulos.github.io/GLMMadaptive/articles/Goodness_of_Fit.html. The GazeVisToolbox to import eye data into matlab can be found at: https://github.com/djangraw/GazeVisToolbox.
