# Supplementary material for: Confidence in subjective pain is predicted by reaction time during decision making
Source: Sci Rep. 2020 Dec 7;10:21373. doi: 10.1038/s41598-020-77864-8 (PMC7721875; doi:10.1038/s41598-020-77864-8)
Supplement: Supplementary file 1 — Supplementary Information. [file 41598_2020_77864_MOESM1_ESM.docx]

Supplementary Information

To accompany “Confidence in subjective pain is predicted by reaction time

during decision making”

Troy C. Dildine, Elizabeth A. Necka, Lauren Y. Atlas

Supplementary Methods

*Pain rating scale instructions.* Prior to the task, participants were instructed about the pain rating scale and the three-second looking period. The same information was displayed on a screen in front of them and read aloud by the experimenter. Subjects were told “First, you will see just the scale. You will have 3 seconds to think about your rating. Then an arrow will appear. Use the mouse to move the arrow to the point on the scale that corresponds to your pain on that trial. You will then state your rating aloud. Feel free to use decimals when you rate the pain aloud. Clicking to the left side of a box means a low value (for example, 5.1) whereas clicking on the right side of the box means it’s more painful (for example, 5.9).” They were then given time to try using the mouse to click and record a rating. The experimenter then explained the pain scale. Participants were told “If you feel no change at all, you should rate that a 0. Click to the left of the scale, below the number 1. If the heat is warm, but not at all painful, you should rate that a 1. If the heat is slightly painful, that would be a 2. A 5 means the heat was moderately painful. An 8 means the most pain you’d be willing to tolerate. If you were holding a hot cup of coffee, at 8 you could still hold on it, but beyond 8, you’d have to put it down. Finally, a 10 is the most imaginable pain. We do not want to reach that rating today.” Finally, the experimenter provided instructions for trials that were intolerable. Participants were told, “If the heat is more than you are willing to tolerate, let us know that it was greater than 8 (e.g., 8.5 or 9), and we will not apply that temperature at that site again. You can also let us know and we will stop the stimulus immediately, or you can remove the thermode from your arm if you must.”

*Instructions for rating uncertainty.*  After explaining the pain rating scale, the experimenter displayed the uncertainty scale. Participants were told, “If you feel certain about the number you rated for pain, you will rate towards completely certain or towards zero on the scale. However, if you don’t feel certain about the number you rated for pain, you will rate towards completely uncertain or towards 100 on the scale.” Participants were then asked, “If you rate pain as an 8, maximum tolerable pain, and feel very certain that you cannot experience any more heat but can experience that level again, how would you rate uncertainty?” Participants provided responses and the experimenter confirmed that the participant had recorded a value towards zero, completely certain, or reiterated the anchors until the participant understood the scale. The experimenter then asked, “If you rate pain as a 2 [pain threshold] but were going back and forth about rating a stimulus as painful or not, how would you rate your uncertainty?” Participants provided responses using a mouse and the experimenter reviewed the response and reiterated the scale to ensure the rating moved towards completely uncertain (i.e. towards 100).

*Corrections for confidence ratings below 0 and above 100*. Pain and confidence ratings were transformed from raw pixel values to appropriate ratings by accounting for the screen position of the visual analogue scale. If a confidence rating was coded below zero or above 100 (i.e. the participant clicked slightly to the left or right of the scale), the mouse position was verified, and the rating was recoded as zero or 100 accordingly. The zero was of particular interest for our analyses, as it determined what entered the logistic portion of the model (to identify associations between completely certain and uncertain trials). We determined that a response of zero on the scale could be captured by 11.15 pixels or 0.8% of the scale (8 pixels for width of scale border and 3.15 pixels for width of arrow). Therefore, any response below 0.8 was coded as zero in our two-part mixed model.

*Initial linear mixed models.* We initially ran multilevel models to analyze our data (using the glmfit_multilevel.m function, https://github.com/canlab/CanlabCore) as independent and dependent variables were measured over time, within individuals. We used separate models for response time and number of fixations. In each model, uncertainty was modeled as a dependent variable and time (trial number) and temperature were added as within-subject’s covariates of interest. We also modeled reliability as a second-level between-subjects moderator to determine whether associations were related to performance across individuals.

To verify our model met model assumptions, we visualized residuals from our multilevel models via a qq-plot (see Supplemental Figure 1). The qq-plot showed skewness, which aligned with the zero-inflation present in the uncertainty data. In response to the model’s failure to meet model assumptions, we transformed the uncertainty data. We first attempted to apply a log transformation on the uncertainty data and followed that with a square root transformation (via the standard log and sqrt functions in Matlab). We visualized our level-one residuals via a qq-plot (see Supplemental Figure 2) and identified persistent skewness in the residuals in both models. In response, we ran a two-part multilevel model to separate the zero and non-zero uncertainty data. We visualized our residuals via a qq-plot (see Supplemental Figure 3) and confirmed that the distribution was appropriate for our data.

*Two-stage simple statistics approach to the two-part multilevel model.* To validate results from the two-part multilevel models, which is still relatively new in R, we used a two-stage summary statistic approach (Gelman 2005) to run our multilevel models. This approach is similar to the summary statistics approach used in fMRI analyses, in which regression models are run on trial level predictors and outcomes within each individual, then analyses are conducted across subjects by using beta estimates from the individual participants’ regression models as the dependent variable.

We ran models comparable to our models of best fit for the two-part model (i.e., including reliability and time in our model for number of fixations, and including no additional predictors for the reaction time model).

Our subject-level linear models included

1) number of fixations: Y_ij_(uncertainty) = (β_0j_ + β_1j_ fixations_ij_ + β_2j_time_ij_) + r_ij_

2) reaction time: Y_ij_(uncertainty) = (β_0j_ + β_1j_ reaction time_ij_) + r_ij_

and our subject-level logistic models included

1) number of fixations: Y_ij_(uncertainty = 1) = (β_0j_ + β_1j_ fixations_ij_ + β_2j_time_ij_) + r_ij_

2) reaction time: Y_ij_(uncertainty = 1) = (β_0j_ + β_1j_ reaction time_ij_) + r_ij_

where i is the trial, j is the individual participant, β_0j_ is the intercept for an individual, β_1j_ is the slope between the n-th predictor and the dependent variable, and r is our error term. The linear regressions applied only to trials rated with uncertainty and included 61 individuals who had at least four trials of uncertainty. The logistic regressions used a binary variable to code for zero and non-zero uncertainty and had the same restrictions as the two-part model (at least four trials of uncertainty and certainty); however, the binomial family in R tries to predict a positive case (1) rather than zero. Thus, we expect the opposite sign in the resulting beta-coefficients from the logistic regressions compared to the two-part model. Main effects across subjects were tested by submitting individual beta-estimates for each predictor to a one-sample t-test (using the t.test function in R) to test if the average beta estimate was significantly different from zero. We also evaluated reliability (i.e., goodness of fit between stimulus temperature and self-reported pain) as a potential subject-level moderator in the number of fixations model, as we did in our two-part model. We ran a linear regression between reliability and the individual-level beta-estimates for both the linear and logistic models. We report the results separately for the logistic and linear portions of our two-stage models in the Supplementary Results section.

*Validating two-part model with separate single-part multilevel linear and logistic models.* To further validate our results, we tried running the two-part model as separate multilevel linear and logistic models with single-part multilevel functions. We ran a multilevel linear model with the lmer function from the lme4 package (v 1.1-23) on non-zero uncertainty trials (comparable to the linear portion of the two-part model) and we ran a multilevel logistic model with the glmer function from the lme4 package (v 1.1-23) to compare zero and non-zero uncertainty trials (comparable to the logistic portion of the two-part model). The linear models were run across all individuals with at least 4 trials of uncertainty (reaction time n = 65; number of fixations n = 61) and the logistic models were run across all individuals with at least 4 trials of uncertainty and complete certainty (reaction time n = 37; number of fixations n = 35). We used the same parameters as in the two-part model:

1. Linear model for reaction time:

**Uncertainty_ij_ > 0 = (γ_00_ + γ_10_ReactionTime_ij_ ) + (*u*_0j_) + r_ij_**

1. Logistic model for reaction time:

**Uncertainty_ij_  = 1 = (γ_00_ + γ_10_ReactionTime_ij_ ) + (*u*_0j_) + r_ij_**

**ln**

**1 – Uncertainty_ij_ = 1**

1. Linear model for number of fixations:

**Uncertainty_ij_ > 0 = (**γ**_00_ +** γ**_10_FixationNumber_ij_ +** γ**_10_time_ij_ +** γ**_01_R^2^_j_ +**

**γ_11_FixationNumber_ij_γ_01_R^2^_j_ ) + (*u*_0j_ ) + r_ij_**

1. Logistic model for number of fixations:

**Uncertainty_ij_  = 1 = (**γ**_00_ +** γ**_10_FixationNumber_ij_ +** γ**_10_time_ij_ +** γ**_01_R^2^_j_ +**

**ln**

**1 – Uncertainty_ij_ = 1** γ**_11_FixationNumber_ij_**γ**_01_R^2^_j_ ) + (*u*_0j_ ) + r_ij_**

However, note that the glmer model also estimates the likelihood of a positive case (1) rather than zero, which the two-part model estimates. Thus, we expect the sign to be in the opposite direction for the glmer models compared to the results from the logistic portion of the two-part model.

Supplementary Results

*Correlational analyses restricted to painful trials.* Results in the main manuscript include all trials. Here, we measured associations restricted to trials that were rated as painful (i.e. pain greater than or equal to 2). Across participants, we still observed a positive association between uncertainty and reaction time (*M*_rho_ = 0.14, *SD*_rho_ = 0.24, *t*(70) = 4.70, *p* < 0.001, CI [.08, .19]), such that participants took longer to rate pain when they were more uncertain. There was a negative association between uncertainty and time (*M*_rho_ = -0.11, *SD*_rho_ = 0.37, *t*(70) = -2.37, *p* = 0.02, CI [-.19, -.02]), such that individuals reported less uncertainty on later trials. Again, uncertainty was not associated with number of fixations (*p* > 0.6). However, in contrast to our findings across all trials, temperature was negatively related to uncertainty when we measured associations within painful trials only (*M*_rho_ = -0.09, *SD*_rho_ = 0.30, *t*(70) = -2.43, *p* = 0.02, CI [-.16, -.02]) such that uncertainty was greater at lower temperatures. Findings of reduced uncertainty with higher temperatures are in line with previous research which suggests increased sensory information increases confidence in perceptual decisions.

*Two-part model restricted to painful trials: Reaction time.* The following results are based on a two-part model with reaction time that was restricted to trials rated as painful (i.e., trials rated at or above a 2 on the pain scale). The logistic portion of the model revealed a negative effect of reaction time in predicting certainty (β_1Logistic_ = -0.59, Odds: 0.55, *SEM* = 0.20, *z* = -2.95, *p* = 0.003), such that increasing log-normal reaction time by one log-unit (i.e., slowing the response) decreased the odds of being certain by 45%. Similar to our findings across all trials, longer reaction times were associated with lower odds of expressing complete certainty. The linear portion of the two-part model revealed a positive effect of log-normal reaction time on log-normal uncertainty (β_1Linear_ = 0.35, *SEM* = 0.11, *z* = 3.1, *p* = 0.002). Similar to our findings across all trials, slower ratings were associated with higher uncertainty.

*Two-part model restricted to painful trials: Number of fixations.* In the logistic portion of our model restricted to painful trials, we observed an interaction between number of fixations and reliability (β_1Logistic_ = -1.14, Odds: 0.32, *SE* = 0.39, *z* = -2.98, *p* = .004), such that a one unit increase in number of fixations is associated with a 1% decrease in odds of being certain when reliability is average, but for every unit increase in reliability the effect of number of fixations on odds of being certain increases by 68%. These results mimic the results across all trials, such that for participants who exhibited greater reliability in their association between pain and temperature, more fixations were associated with higher odds of uncertainty, whereas for participants who exhibited less reliability, more fixations were associated with higher odds of certainty. Similar to our results across all trials, there was no additional association between uncertainty and fixation in the linear part of the model (p > 0.1), there was no relationship with time in the logistic portion of the model (p > 0.1), and there was a negative association between time and uncertainty (β_1Linear_ = -0.02, *SEM* = 0.008, *z* = -3.2, *p* = 0.002) in the linear portion of the model, such that participants reported more certainty later in the task.

*Two-part model with reaction time, including time and the interaction between time and reaction time as fixed effects.* The following results are based on a two-part model with reaction time that included time (trial number) and an interaction term between time and reaction time, to determine if time in the task (which negatively associated with reaction time in the correlational analyses) impacted any of the results from the main manuscript. The logistic portion of the model revealed a similar negative effect of reaction time in predicting certainty (β_1Logistic_ = -0.54, Odds: 0.58, *SEM* = 0.19, *z* = -2.87, *p* = 0.004), such that increasing log-normal reaction time by one log-unit (i.e., slowing the response) decreased the odds of being certain by 42%. In other words, longer reaction times were associated with lower odds of expressing complete certainty. There was no interaction between time (trial number) and reaction time in the logistic portion of the two-part model (*p* > 0.7), suggesting the logistic association between reaction time and confidence holds throughout the task. The linear portion of the two-part model revealed a similar positive effect of log-normal reaction time on log-normal uncertainty (β_1Linear_ = 0.33, *SEM* = 0.11, *z* = 2.90, *p* = 0.004) such that slower ratings were associated with higher uncertainty. There was no interaction between time (trial number) and reaction time in the linear portion of the two-part model (*p* > 0.75), suggesting the linear association between reaction time and confidence holds throughout the task.

*Two-stage multilevel model: Reaction time.* We observed a marginal difference from zero in subject-level betas from both the logistic (*t*(36) = 2.02, CI = [-0.004, 1.88], *p* = 0.051) and linear (*t*(64) = 2.15, CI = [0.04, 1.11], *p* = 0.04) regressions between reaction time and uncertainty. Although the results from both models are not significant based on Bonferroni corrections, they trend in the direction of the two-part model. The full results are located in Supplemental Table S4.

*Two-stage multilevel model: Number of fixations.* In estimating the direct association between number of fixations and uncertainty, we observed similar results to the two-part model. There was no difference from zero in the subject-level betas from the logistic (*p* = 0.84) nor the linear regressions (*p* = 0.18) between uncertainty and number of fixations. Similarly, average subject-level beta estimates from the linear regressions did differ from 0 (*t*(60) = -2.9, *p* = 0.005, CI [-0.05, -0.009]) between time and uncertainty but did not from the logistic regressions (*p =* 0.82). The full results are located in Supplemental Table S5.

Consistent with the interaction between reliability and number of fixations in the two-part model, we observed a linear association between the subject-level betas from the logistic regressions for number of fixations with reliability via the simple statistics approach (β = 1.89, *SE* = 0.50, *t(35)* = 2.54, *p* < 0.001). We did not observe an association between the subject-level betas from the linear regressions (*p* = 0.54) and reliability, also comparable to the results from the two-part model. The full results are located in Supplemental Table S6.

*Single-part multilevel linear and logistic models: Reaction Time.* We observed a difference between reaction time for certain and uncertain trials via glmer (β_1Logistic_ = 0.55, Odds: 1.73, *SEM* = 0.18, *z* = 3.1, *p* = 0.002). This result, such that slower reaction time increases the odds of an uncertain trial, validates the outcome from the two-part model that quicker reaction times are associated with certain trials. Furthermore, we observed a linear association between reaction time and uncertainty via lmer (β_1Linear_ = 0.25, *SEM* = 0.05, *t* = 4.8, *p* < 0.001), that also replicated the result from the two-part model (slower responses positively associated with uncertainty). The full results are located in Supplemental Table S7.

*Single-part multilevel linear and logistic models: Number of fixations.* In the non-linear multilevel model via glmer, we replicate the interaction between number of fixations and the reliability of an individual (β_1Logistic_ = 1.09, Odds: 2.97, *SEM* = 0.33, *z* = 3.3, *p* = 0.001) observed in the two-part model. We did not find differences for any of the remaining factors (all *p*’s > 0.4), which also replicates outcomes from the two-part model. In the linear multilevel model via lmer, we observed a linear effect of time on uncertainty (β_1Linear_ = -0.02, *SEM* = 0.004, *t* = -4.2, *p* < 0.001). We observed no other linear associations with uncertainty (all *p*’s > 0.3). These results also replicate the outcomes from the two-part model. For the full results, see Supplemental Table S8.

Supplementary Tables

Supplemental Table S1. Spearman’s Correlations with uncertainty ^1^.

|  | ***Rho*** | ***SD*** | ***t*** | ***p*** |
| --- | --- | --- | --- | --- |
| *Time* | -0.08 | 0.34 | -2.05 | 0.04 |
| *Temperature* | 0.02 | 0.28 | 0.62 | 0.53 |
| *Reaction Time* | 0.12 | 0.24 | 4.38 | < 0.001 |
| *Number of Fixations* | 0.03 | 0.27 | 0.04 | 0.97 |

^1^ Rho coefficient and standard deviations for Spearman’s correlations between independent variables (listed in the first column) and explicit confidence are listed. A t statistic and p-value are reported for one-sample t-tests based on rho coefficients from the within-subject correlations. Temperature, time, and reaction time each included 72 within-subject Spearman’s correlations and number of fixations included 66 within-subject Spearman’s correlations.

Supplemental Table S2. *Comparisons of reaction time models via likelihood ratio tests (LRT)^2^.*

| **Models** | **Deviance** | **LRT**  **Chi-Squared** | ***p*** | | **Reduced Form Equations** | |
| --- | --- | --- | --- | --- | --- | --- |
|  |  |  | |  | |  |
| 1) **Reaction time predicting uncertainty with a random intercept^3^**  2) Adding R^2^ as fixed effect  3) Adding R^2^*RT interaction  4) Adding in time as a covariate  5) Adding in temperature  as a covariate  6) Adding in random effect for Reaction time | **2304.33**  2303.35  2303.32  2301.25  2301.98  2299.52 | -    χ²(2)= 0.98^4^    χ²(4)= 1.01  χ²(2)= 3.08  χ²(2)= 2.35  χ²(2)= 4.81 | -  0.61  0.91  0.21  0.31  0.09 | | **Uncertainty_ij_ = (γ_00_ + γ_10_ReactionTime_ij_ )**  **+ (*u*_0j_) + r_ij_**  Uncertainty_ij_ = (γ_00_ + γ_10_ReactionTime_ij_ + γ_01_R^2^_j_ ) + (*u*_0j_ ) + r_ij_  Uncertainty_ij_ = (γ_00_ + γ_10_ReactionTime _ij_ + γ_01_R^2^_j_ + γ_11_FixationNumber_ij_γ_01_R^2^_j_ ) + (*u*_0j_ ) + r_ij_  Uncertainty_ij_ = (γ_00_ + γ_10_ReactionTime_ij_ + γ_10_time_ij_) + (*u*_0j_ ) + r_ij_  Uncertainty_ij_ = (γ_00_ + γ_10_ ReactionTime _ij_ + γ_10_temperature_ij_) + (*u*_0j_) + r_ij_  Uncertainty_ij_ = (γ_00_ + γ_10_ReactionTime _ij_ ) + (*u*_0j_ + *u*_1j_ReactionTime_ij_ ) + r_ij_ | |

^2^ We ran the simplest model with GLMMadaptive and added fixed terms one by one, to determine if they improved the fit of the model. We included reliability (R^2^) as a potential factor of interest and looked at its interaction with reaction time. As time and temperature were covariates of interest, we did not look at their interaction with reaction time. Finally, we tried adding a random effect for reaction time. No addition significantly improved model fit. Therefore, we chose the first model for our analyses. The reduced form equation is showing the linear portion of the model, the logistic portion includes the same fixed and random effects terms.

^3^ The GLMMadaptive package requires that a random intercept be included in all models.

^4^ As the model contains both a linear and logistic portion, each additional parameter creates two estimates (one for the linear and one for the logistic). Therefore, we set parameter change to two in estimating alpha in our chi-square metric.

Supplemental Table S3. *Comparisons of fixation models via likelihood ratio tests (LRTs)^5^.*

| **Models** | **Deviance** | **LRT**  **Chi-Squared** | ***p*** | | **Reduced Form Equations** | |
| --- | --- | --- | --- | --- | --- | --- |
|  |  |  | |  | |  |
| 1) Number of fixations predicting uncertainty with a random intercept  2) Adding R2 as fixed effect (compared to Model 1)  3) Adding R2*Fixation interaction (compared to Model 1)  **4) Adding in time (covariate) as a fixed effect (compared to Model 3)**  5) Adding in temperature  (covariate) as a fixed effect (compared to model 4)  6) Adding in a random effect for number of fixations (compared to model 4) | 2163.46  2162.48  2148.90  **2141.75**  2140.80  2140.29 | -    χ²(2)= 0.98    χ²(4)= 14.56  χ²(2)= **7.15**  χ²(2)= 0.95  χ²(2)= 1.46 | -  0.61      0.005  **0.03**  0.62  0.48 | | Uncertainty_ij_ = (γ_00_ + γ_10_FixationNumber_ij_) + (*u*_0j_) + r_ij_  Uncertainty_ij_ = (γ_00_ + γ_10_FixationNumber_ij_ + γ_01_R^2^_j_) + (*u*_0j_) + r_ij_  Uncertainty_ij_ = (γ_00_ + γ_10_FixationNumber_ij_ + γ_01_R^2^_j_ + γ_11_FixationNumber_ij_γ_01_R^2^_j_) + (*u*_0j_) + r_ij_  **Uncertainty_ij_ = (γ_00_ + γ_10_FixationNumber_ij_ + γ_10_time_ij_ + γ_01_R^2^_j_ + γ_11_FixationNumber_ij_γ_01_R^2^_j_) + (*u*_0j_) + r_ij_**  Uncertainty_ij_ = (γ_00_ + γ_10_FixationNumber_ij_ + γ_10_time_ij_ + γ_10_temperature_ij_ + γ_01_R^2^_j_ + γ_11_FixationNumber_ij_γ_01_R^2^_j_) + (*u*_0j_) + r_ij_  Uncertainty_ij_ = (γ_00_ + γ_10_FixationNumber_ij_ + γ_10_time_ij_ **+** γ_01_R^2^_j_ + γ_11_FixationNumber_ij_γ_01_R^2^_j_) + (*u*_0j_ + *u*_1j_FixationNumber_ij_) + r_ij_ | |

^5^ We ran the simplest model with GLMMadaptive and added fixed terms one by one to determine if they improved the fit compared to the simplest model (Model 1). We included reliability (R^2^) as a potential factor of interest and looked at its interaction with reaction time. Including the interaction term improved the model significantly. We included time as a covariate and note that this significantly improved the model compared to our previously best fit model, Model 3. We added temperature as a covariate of interest, but this did not perform significantly better than Model 4. Finally, we tried adding a random effect for number of fixation, but this addition did not significantly improve model fit compared to Model 4. Therefore, we chose the fourth model for our analyses. The reduced form equation is showing the linear portion of the model, the logistic portion includes the same fixed and random effects terms.

Supplemental Table S4. *Two-stage simple statistics model between Uncertainty and Reaction Time^6^*

|  | **Variables** | ***M _β_*** | **CI** | ***t*** | ***p*** |
| --- | --- | --- | --- | --- | --- |
| *Reaction Time* | *Logistic* | 0.57 | [0.04,1.11] | 2.02 | 0.051 |
|  | *Linear* | 0.94 | [-0.004, 1.88] | 2.15 | 0.04 |

^6^ Logistic and linear regressions were performed between uncertainty and reaction time for each individual. A random intercept was included to mimic the two-part model. One sample t-tests were performed across the subject-level betas for both the logistic and linear models. The logistic regression included 37 individuals (all individuals with at least 4 trials of uncertainty and certainty) and the linear regression included 65 individuals (all individuals with at least 4 trials of uncertainty).

Supplemental Table S5. *Association between uncertainty and number of fixations via one-sample t-tests based on Two-stage simple statistics^7^*

|  | **Variables** | ***M _β_*** | **CI** | ***t*** | ***p*** |
| --- | --- | --- | --- | --- | --- |
| *Logistic* | *Number of Fixations* | -0.02 | [-0.18,0.15] | -0.20 | 0.84 |
|  | *Time* | -0.006 | [-0.06, 0.05] | -0.24 | 0.82 |
| *Linear* | *Number of Fixations* | -0.04 | [-0.09, 0.02] | -1.35 | 0.18 |
|  | *Time* | -0.03 | [-0.05, -0.008] | -2.90 | 0.005 |

^7^ *One-sample t-tests on subject-level betas between Uncertainty and Number of Fixations.* One sample t-tests were run across subject-level betas from within-subject linear and logistic regressions to determine associations between uncertainty and number of fixations. The regressions were set with the same parameters as the two-part model. The logistic regression included 35 individuals and the linear regression included 61 individuals.

Supplemental Table S6. *Association between reliability and mean effect of fixations on uncertainty based on Two-stage simple statistics^8^*

|  | **Variables** | **β** | ***SE*** | ***t*** | ***p*** |
| --- | --- | --- | --- | --- | --- |
| *Logistic* | *Intercept* | -0.02 | 0.07 | -0.38 | 0.71 |
|  | *Association*  *with R^2^* | 1.89 | 0.50 | 2.54 | < 0.001 |
| *Linear* | *Intercept* | -0.05 | 0.03 | -1.94 | 0.06 |
|  | *Association*  *with R^2^* | 0.10 | 0.16 | 0.61 | 0.54 |

^8^ *Moderation by Reliability between Uncertainty and Number of Fixations.* A linear regression was run between reliability and the subject-level beta estimates for both the linear and logistic models for number of fixations. Comparable to the numbers for the one sample t-tests, the model including subject-level betas from the logistic regressions included 35 individuals and the model including subject-level betas from the linear regression included 61 individuals.

Supplemental Table S7. *Association between reaction time and uncertainty based on separate single-part multilevel linear and logistic model^9^*

|  | **Variables** | ***β*** | ***SE*** | **Test Statistic^10^** | ***p*** |
| --- | --- | --- | --- | --- | --- |
| *Logistic* | *Intercept* | -0.05 | 0.16 | -0.36 | 0.75 |
|  | *Reaction Time* | 0.55 | 0.18 | 3.11 | 0.002 |
| *Linear* | *Intercept* | 2.20 | 0.09 | 23.98 | < 0.001 |
|  | *Reaction*  *Time* | 0.25 | 0.05 | 4.81 | < 0.001 |

^9^ *Commonly used multilevel linear and logistic models: Reaction Time.* We ran a multilevel linear model with the lmer function on non-zero uncertainty trials and we ran a multilevel nonlinear model with the glmer function to compare zero and non-zero uncertainty trials. The linear model was run across all individuals with at least 4 trials of uncertainty (n = 65) and the non-linear model was run across all individuals with at least 4 trials of uncertainty and complete certainty (n = 37). Each model included an intercept and reaction time as a predictor.

^10^ The logistic multilevel model, via glmer, reports z-values and the linear multilevel model, via lmer, reports t-values.

Supplemental Table S8. *Associations between uncertainty and fixation number based on separate single-part multilevel linear and logistic models: Number of fixation^11^*

|  | **Variables** | ***β*** | ***SE*** | ***Test Statistic^12^*** | | | ***p*** |
| --- | --- | --- | --- | --- | --- | --- | --- |
| *Logistic* | *Intercept* | -0.08 | 0.17 | | -0.48 | 0.63 | |
|  | *Number of Fixations* | 0.01 | 0.05 | | 0.25 | 0.80 | |
|  | *Reliability* | -0.92 | 1.19 | | -0.77 | 0.44 | |
|  | *Time* | -0.009 | 0.01 | | -0.75 | 0.45 | |
|  | *Number of fixations * Reliability* | 1.09 | 0.33 | | 3.30 | 0.001 | |
| *Linear* | *Intercept* | 2.20 | 0.10 | | 22.74 | < 0.001 | |
|  | *Number of Fixations* | 0.10 | 0.01 | | 0.64 | 0.52 | |
|  | *Reliability* | 0.49 | 0.58 | | 0.85 | 0.40 | |
|  | *Time* | -0.02 | 0.003 | | -4.21 | < 0.001 | |
|  | *Number of fixations * Reliability* | 0.06 | 0.08 | | 0.69 | 0.49 | |

^11^ *Commonly used multilevel linear and logistic models: Number of fixations.* We ran a multilevel linear model with the lmer function on non-zero uncertainty trials and we ran a multilevel nonlinear model with the glmer function to compare zero and non-zero uncertainty trials. The linear model was run across all individuals with at least 4 trials of uncertainty (n = 61) and the non-linear model was run across all individuals with at least 4 trials of uncertainty and complete certainty (n = 35). Each model included an intercept, number of fixations, reliability, time, and the interaction between fixation and reliability as predictors.

^12^ The logistic multilevel model, via glmer, reports z-values and the linear multilevel model, via lmer, reports t-values.

Supplementary Figures

Supplemental Figure S1. QQ-plots of residuals from original multilevel models


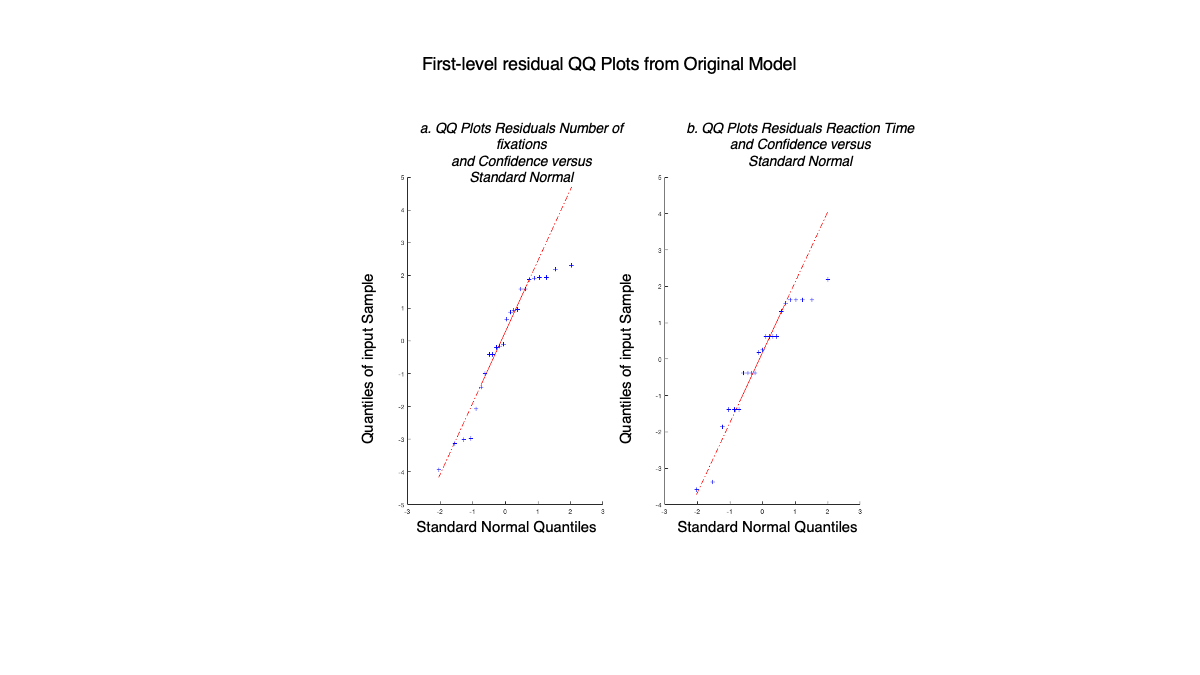


Supplemental Figure S2. *QQ Plot of Residuals from multilevel models with transformed uncertainty*


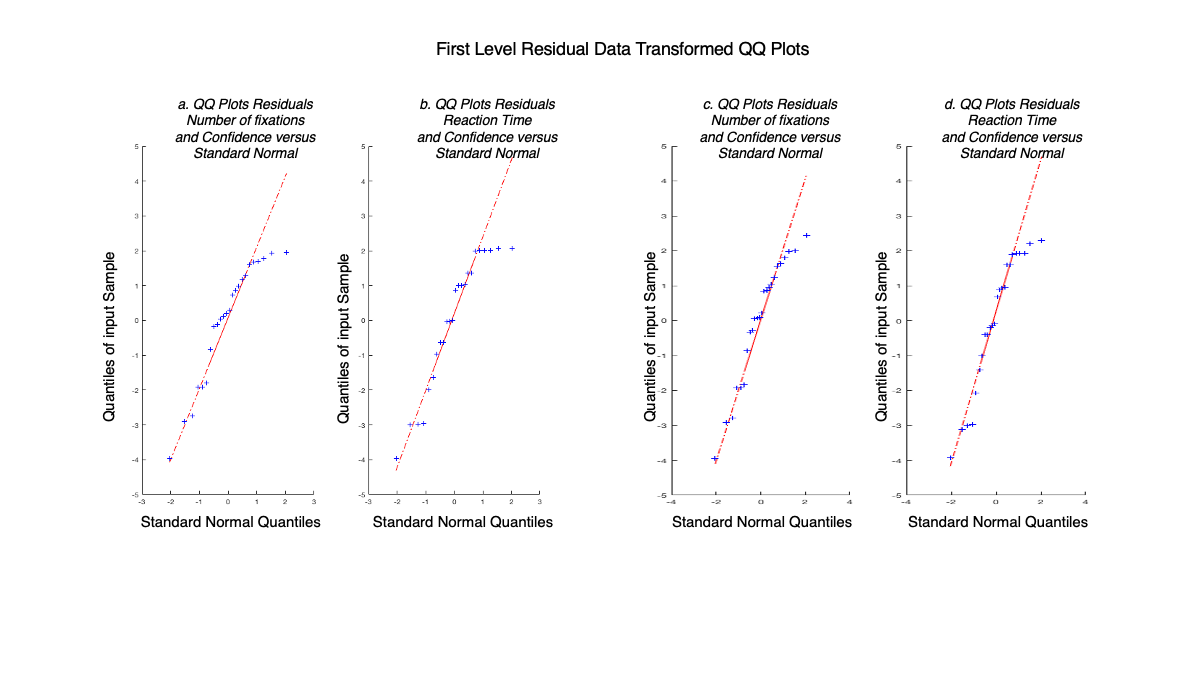


Supplemental Figure S3.  *QQ Plots of the residuals* from the two-part models


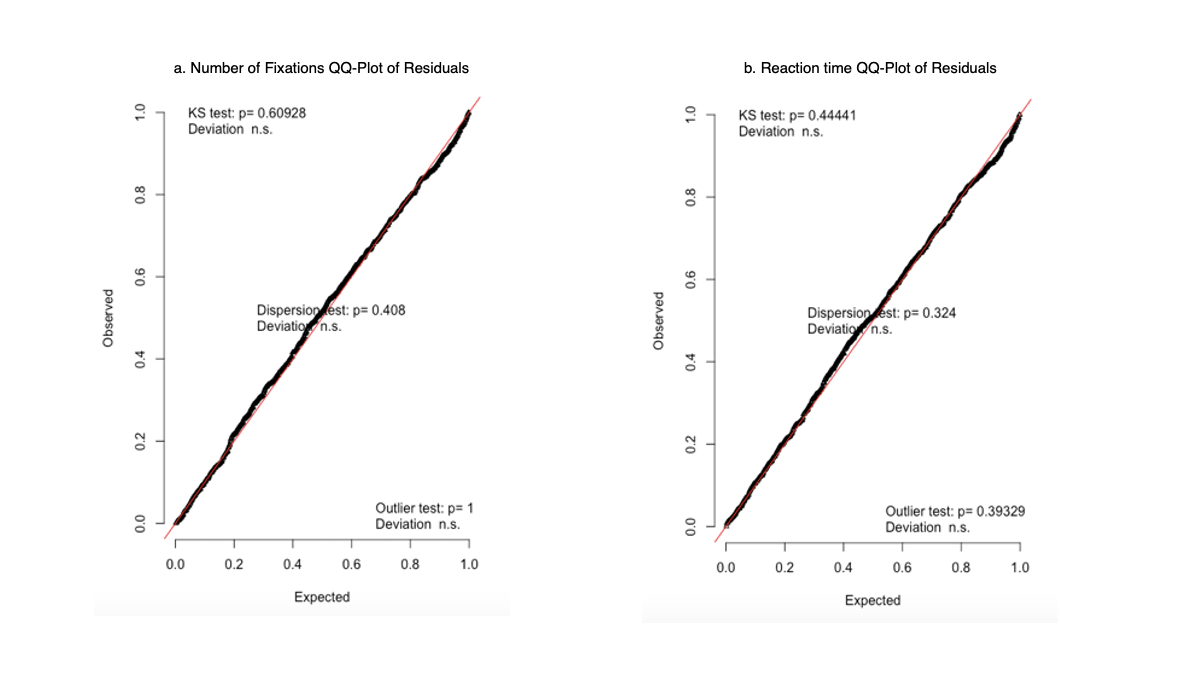


Supplementary Figures Legend

Supplemental Figure S1: *QQ Plot:*  We ran linear models to look at the effect of reaction time (left plot) and number of fixations (right plot) on uncertainty. The resulting residuals were set against the Standard Normal via QQ plots. The plot shows curvature near the end of the line, a right-skewed tail, highlighting zero-inflated data.

Supplemental Figure S2: A. *QQ Plot of Log-Transformed data:*  We log-transformed uncertainty and ran linear multilevel models to predict log-transformed uncertainty in response to our original model failing to meet assumptions. The resulting residuals were set against the Standard Normal via QQ plots (RT to the left and number of fixations to the right). The QQ plots show curvature at the end of the line, suggesting persistent extreme values. B. *Square root transformation QQ Plot*: We square root transformed uncertainty and ran linear multilevel models between square root transformed uncertainty and our predictors. The resulting QQ plots (RT to the left and number of fixations to the right), show slightly better fits, but still curvature at the end of the line from our zero-inflated data.

Supplemental Figure S3: A. *Two-part model QQ Plots*, *Reaction Time:* We used a modification of the DHARMa package in R to visualize the residuals from our two-part model (run with the GLMMadaptive package). The line shows no deviation from the expected residuals, indicating that our model fit the data appropriately and that we met model assumptions. *B. Two-part model QQ Plots, Number of Fixations.* Again, we find no deviation from our expected line, suggesting that our model met model assumptions.
